# Cardio-metabolic parameters are associated with genetic admixture estimates in a pediatric population from Colombia

**DOI:** 10.1186/s12863-016-0402-5

**Published:** 2016-06-27

**Authors:** Angélica M. Muñoz, Claudia M. Velásquez, Gabriel Bedoya

**Affiliations:** Research Group on Food and Human Nutrition, Universidad de Antioquia (UdeA), Calle 70 No. 52-21, Medellín, Colombia; Research Group on Molecular Genetic (GENMOL), Universidad de Antioquia (UdeA), Calle 70 No. 52-21, Medellín, Colombia; Laboratorio 413, Sede de Investigación Universitaria (SIU), Universidad de Antioquia (UdeA), Calle 70 No. 52-21, Medellín, Colombia

**Keywords:** Ancestry, Admixed population, Cardio-metabolic parameters, Youth

## Abstract

**Background:**

There are different genetic patterns for cardio-metabolic parameters among different populations. Additionally, it has been found that ancestral genetic components (the proportion of Amerindian, European and African) in admixed Latin American populations influence an individual’s susceptibility to cardio-metabolic disorders. The aim of this study was to evaluate the effect of ancestral genetic composition on a series of cardio-metabolic risk factors in a young admixed population from Colombia.

**Results:**

In a sample of 853 Colombian youth, 10 to 18 years old, the mean European contribution was 66.6 % (range: 41–82 %), the mean African contribution was 14 % (range: 4–48 %), and the mean Amerindian contribution was 19.4 % (range: 10–35 %) using a panel of 40 autosomal ancestry-informative markers (AIMs). We assessed the degree of association between ancestral African, Amerindian and European genetic components and measures of body mass index, waist circumference, fasting glucose, fasting insulin, insulin resistance, triglycerides, high-density lipoprotein, and systolic and diastolic blood pressure. Two of the nine measures assessed presented a nominal significant association with ancestral components after adjusting for confounding variables: triglyceride levels were associated with the Amerindian component (OR = 1.06, 98.3 % CI = 1.01–1.11, *P* = 0.002) and systolic blood pressure was associated with the European component (OR = 0.93, 98.3 % CI = 0.87 to 0.99, *P* = 0.008) and the African component (OR = 1.07, CI = 1.01–1.14 *P* = 0.008), although it was not significant following a global Bonferroni correction. Additionally, insulin levels and insulin resistance showed associations with the African component.

**Conclusions:**

Our findings support the idea that an Amerindian ancestral component may act as a risk factor for high triglyceride levels. In addition, an African ancestral component confers a risk for high systolic blood pressure, and a European ancestry serves as a protective factor for this condition in a young admixed population from Colombia. However, these results should be confirmed in a larger population.

**Electronic supplementary material:**

The online version of this article (doi:10.1186/s12863-016-0402-5) contains supplementary material, which is available to authorized users.

## Background

The genomic composition of Latin American (LA) populations is the result of a tri-ethnic genetic admixture that occurred during the colonization of the American continent. This process has been documented with historical data and initially corroborated via lineage-based tests, which target mitochondrial DNA and the non-recombining Y chromosome [1, 2]. Subsequently, with the discovery of an autosomal marker-based test to detect sequences with large differences in their allelic frequencies among continental populations (δ), called ancestry-informative markers (AIMs), it has been possible to infer the proportions of each of the three ancestral components in an admixed LA genome [3, 4]. Using AIMs, the ancestral genetic compositions of several LA populations, including the Colombian population, have been determined [3, 5]. The results show that the majority of LA populations were founded by a complex admixture process among three continental populations (Amerindian, African and European), although the proportions vary among – and even within – countries [6].

Knowledge of the ancestry of Latin American populations has different applications, ranging from forensic and anthropological uses to informing biomedical sciences, and can be especially useful in the identification of gene variants associated with both infectious and complex diseases [7, 8].

Using genetic admixture as a tool to identify gene variants involved in complex diseases requires differences in allelic frequencies among the parental population and recent occurrence of admixture mating among those parental populations. The genetic admixture is a factor that influences the allelic frequencies in a population and this, in part, contribute to explaining the differences observed in the epidemiology of certain diseases in admixed population regarding parental populations. These two conditions are fulfilled in the Colombian population because the diseases involved in cardio-metabolic disorders, such as obesity, type 2 diabetes mellitus (T2D), hypertension and dyslipidemia, have different prevalences in European, African and Amerindian populations [9] and because the demographic history of the genetic admixture is very recent [5]. Globally, the prevalence of overweight and obesity varies across different regions. In adults, it is the highest in the Americas (61.3 %) and is the lowest in Africa (30.8 %). The prevalence of diabetes is the highest in the American/Caribbean region and the lowest in Africa (12.9 % and 3.2 %, respectively), whereas the prevalence of high blood pressure in adults is the highest in Africa (30 %) and is lower in the Americas (18 %). Finally, the prevalence of elevated total cholesterol is the highest in Europe (54 %) and the lowest in Africa (22.6 %) [9, 10] (See Additional file 1: Table S1). Because of this heterogeneity, genetic admixture studies provide a unique opportunity to account for the heterogeneity of population-based differences and to understand the role of genetic factors that contribute to disease risks in admixed populations. However, there have been no studies evaluating the effect of ancestry on cardio-metabolic parameters conducted on people under 18 in the Colombian population. Finally, the identification of the effect of genetic variants in the prevalence of such diseases requires that the effect of variables that interfere with this association, such as socio-economic status, parental education, physical activity and diet, be assessed using a control. Based on the above information, we aimed to evaluate the association between individual and average estimates of genetic ancestry and the anthropometric, biochemical and clinical measurements used to assess cardiovascular risk factors in a Colombian population of admixed youth while adjusting for environmental factors.

## Methods

### Population of the study

A cross-sectional study of 853 unrelated, self-identified healthy volunteers of both sexes between 10 and 18 years old was performed. The population base for subject recruitment was the population examined in following study: “Metabolic Syndrome in overweight youth: Identification of risk factors and evaluation of an intervention.” The participants were affiliated with a company that provides health services in the city of Medellin in northwest Colombia. The exclusion criteria were defined as follows: young people who used corticosteroids or thyroid hormones; those who were hypoglycemic; those diagnosed with diabetes, chronic renal failure, or innate metabolism-related genetic diseases; those with physical or mental disabilities; those who were highly competitive athletes; and those who were pregnant or nursing. This study complied with the Declaration of Helsinki ethical principles for medical research involving human subjects. The researchers complied in all cases with Resolution 8430 of 1993, from the Colombian Ministry of Social Protection.

### General socioeconomic and health information

The participants and their guardians answered a questionnaire and provided general information, including sex, date of birth and socioeconomic status, according to the National Administrative Department of Statistics (*Departamento Administrativo Nacional de Estadística*, DANE), which has established six socioeconomic categories based on housing location, with stratum 1 being the lowest and 6 the highest [11]. For the purpose of this analysis, the data obtained for the socioeconomic strata were classified into three categories: low (1 and 2), middle (3 and 4) and high (5 and 6). Pubertal maturation was based on a self-evaluation performed by the subjects according to the Tanner Sexual Development Stages using pictures or schematic drawings [12, 13]. In addition, information on birth weight and the duration of breastfeeding was obtained.

### Phenotypes

For this study, cardio-metabolic risk factors were defined as follows: waist circumference (WC) ≥ the 90th percentile for the individual’s age and sex (according to values reported in the III National Health and Nutrition Examination Survey (NHANES) for Mexican-American youth) [14]; triglycerides (TGs) ≥110 mg/dL; HDL cholesterol ≤40 mg/dL; systolic or diastolic blood pressure ≥the 90th percentile for the individual’s age, sex and height; and fasting blood glucose ≥100 mg/dL. Additionally, overweight (body mass index (BMI) >85th percentile; insulin >23.0 μU/mL and insulin resistance (IR) (HOMA >3.1) were determined. IR was estimated using the Homeostasis Model Assessment (HOMA-IR) using the HOMA Calculator Version 2.2.2 software from the ©Diabetes Trials Unit from Oxford University. Each of the measures were obtained using standardized protocols and calibrated equipment as referenced by previous studies [15].

### Environmental covariates

*Food intake* was assessed using a 24-hour reminder, which was randomly distributed on different days of the week. A second reminder was given in a random subsample and on non-consecutive days to estimate and adjust for individual variability. The level of physical activity was determined using the University of South Carolina Arnold School of Public Health’s 3-Day Physical Activity Recall (3DPAR) questionnaire, in which participants were asked questions about their physical activity (PA) for the three previous days (two weekdays and one weekend day). For the purposes of this analysis, the data obtained were classified into three final levels: low, moderate, and high [16].

### Genetic ancestry estimation

To infer the individual and average ancestries of the participants, 40 AIMs widely distributed across the genome that show differences in the allele frequencies (higher than 40 % between at least two populations) among the European, Amerindian and African parental populations were selected. AIMs were selected using the previously available population data included in the Marshfield Clinic diallelic insertion/deletion polymorphism database [17], a database of retrotransposon insertion polymorphisms in humans (dbRIP) [18], and from studies that have also characterized markers for Latin American populations [4, 19]. Detailed information about the 40 AIMs are shown in Additional file 1: Table S2 and Table S3. DNA was extracted from peripheral blood using a standard method [20]. The concentration and quality of the samples were determined via spectrophotometry. The AIMs were typified using PCR-RFLP (polymerase chain reaction – restriction fragment length polymorphism) analysis and based on the differences in the amplified fragment sizes (in cases of an In/Del) using capillary electrophoresis in an ABI-PRISM 310 genetic analyzer (Applied Biosystems). Genotype determination was performed using the GeneMapper v 4.0 program.

### Statistical analysis

The assumption of normality of the quantitative variables was evaluated using the Kolmogorov-Smirnov test. The qualitative variables were presented with their frequency distributions; the quantitative variables were summarized according to their median and interquartile ranges. Food consumption data were analyzed based on the Program Evaluation of Dietary Intake (Evaluación de la Ingesta Dietética, EVINDI v4) [21]. Reports of nutrient intake were processed using the program PC SIDE (Personal Computer Version of Software for Intake Distribution Estimation) v 1.0. The allele and genotypic frequencies were calculated with the PLINK v. 1.07 program [22]. Ancestry proportions were calculated with the ADMIXMAP v 3.2 program [23], which uses a frequentist-Bayesian method. The analysis of median differences was performed using Mann-Whitney U or Kruskal-Wallis tests. Individual cardio-metabolic parameters (body mass index, waist circumference, HDL cholesterol, systolic or diastolic blood pressure, fasting blood glucose, and insulin resistance) were coded as binary variables based on the cutoff values described above. Logistic regression analysis was performed using the dichotomous variables as an independent model to assess the effect of each of the genetic ancestries on each cardio-metabolic parameter. Because of the known effects of socioeconomic status, parental education, physical activity and diet on these components, each of these covariates were included in the model as control variables. Analyses were conducted with SPSS (Statistical Package for the Social Sciences) v. 19.0 statistical software and PLINK v. 1.07 [22]. Because all associations assessed were based on a priori hypotheses, both unadjusted and adjusted probability values are reported. The significance test were adjusted by the Bonferroni method, two threshold significance as a *p*-value <0.05/3 = 0.0167 (three ancestry), and *p*-value <0.05/27 = 0.0018 (global correction, three ancestries * nine cardio-metabolic parameters) was considered significant for avoiding the error of multiple testing. The threshold of significance for all other comparisons was *p* <0.05.

## Results

### General characteristics of the study population

A total of 853 young volunteers (10 to 18 years old) from a region of Colombia (Medellín) were included in the study. The average age was 14 ± 2.4 for both male and female. Of the volunteers, 52 % were female, 42 % belonged to the middle socioeconomic stratum, and 54 % were classified as post-pubertal. As for the perinatal and environmental variables, 86 % had an adequate birth weight and 9 % breastfed for less than a month; 29 % of the youth watch TV for >4 h per day; and 33 % engaged in low levels of physical activity. The median energy intake was 2253 kcal/day. Diets consisted of 13 % protein, 55 % carbohydrates and 32 % fat. Table 1 shows the demographic, perinatal, physical activity and food consumption values in relation to each of the cardio-metabolic parameters.

**Table 1:** Characteristics of the study population, stratified according to cardio-metabolic parameters

	WC (cm)	BMI	Fasting Glucose (mg/dL)	Fasting Insulin (μU/mL)	HOMA score	TG (mg/dL)	HDL (mg/dL)	DBP (mmHg)	SBP (mmHg)
Gender^a^									
Females	69.1 (63.4–74.8)**	22.0 (19.7–24.8)	82 (77–88)**	10,2 (7.5–14.7)**	1.3 (1.0–1.9)**	94 (70–135)	51 (43–60)	62 (60–70)**	101 (100–110)**
Males	73.3 (67.2–79.9)	21.9 (19.6–25.0)	85 (80–91)	8.3 (5.8–12.8)	1.0 (0.7–1.6)	91 (63–133)	50 (41,5–58)	70 (61–74)	110 (101–118)
Age group^a^									
10 to 13.9	68.9 (63.0–75.3)**	21.2 (18.6–23.8)**	83 (78–88)**	9.1 (6.4–13.2)*	1.2 (0.8–1.7)	92 (68–131)	52 (44–60)**	62 (60–70)	102 (100–110)**
14 to 18	73.0 (68.1–79.9)	23.0 (20.6–25.9)	85 (80–90)	9.8 (6.9–14.7)	1.2 (0.9–1.9)	93 (65–138)	48 (41–58)	68 (62–73)	110 (101–119)
Socioeconomic Status^b^									
Low	70.0 (63.7–77.2)	21.9 (19.2–25.0)	84 (79–90)*	9.3 (6.4–14.0)	1.2 (0.8–1.8)	98 (70–143)**	50 (42–57)*	64 (60–70)**	105 (100–112)
Medium	70.6 (65.2–76.9)	22.0 (20.0–24.9)	84 (79–90)	9,4 (6,8–13,7)	1.2 (0.9–1.8)	97 (68–136)	50 (42–60)	64 (60–70)	106 (100–115)
High	71.0 (66.0–78.0)	21.9 (19.7–25.1)	81 (77–88)	9,4 (6,4–13,2)	1.2 (0.8–1.7)	78 (59–106)	53 (45–62)	70 (61–70)	105 (100–115)
Pubertal maturation^b^									
Prepubertal	67.3 (62.5–75.8)**	20.5 (18.1–23.5)**	84 (78–89)	8.3 (5.2–13.8)**	1.1 (0.7–1.7)	97 (66–147)	53 (42–60)*	62 (60–70)**	100 (100–109)**
Pubertal	69.5 (63.0–76.1)	21.5 (18.7–24.0)	82 (78–88)	9.2 (7.1–12.8)	1.1 (0.9–1.7)	89 (67–127)	52 (45–60)	64 (60–70)	105 (100–112)
Postpubertal	72.3 (67.4–78.4)	22.9 (20.5–25.5)	84 (79–90	9.9 (6.7–14.1)	1.2 (0.9–1.8)	92 (67–134)	48 (42–58)	66 (60–70)	109 (100–116)
Birth weight, (g)^b^									
Low (<2500)	68,8 (64.5–77.2)	21.2 (19.4–23.8)	82 (75–89)	9.6 (7.5–15.1)	1.2 (0.9–2.0)	92 (70–133)	48 (40–55)	67 (60–70)	106 (100–110)
Adequate (≥2500–4000)	70.2 (64.5–77.0)	21.9 (19.6–24.8)	84 (78–89)	9.2 (6.4–13.5)	1.2 (0.8–1.7)	92 (67–132)	50 (43–59)	64 (60–70)	105 (100–114)
High (>4000)	73.1 (68.1–79.0)	22.5 (20.6–25.9)	82 (78–87)	8.5 (5.8–13.6)	1.1 (0.7–1.8)	83 (63–123)	51 (42–61)	64 (60–70)	109 (100–115)
Duration of breastfeeding, (months)^b^									
>6	70.0 (63.8–76.9)**	21.7 (19.2–24.8)**	84 (79–90)	9.1 (6.3–13.6)*	1.2 (0.8–1.7)**	92 (67–127)	50 (43–60)	64 (60–70)	105 (100–114)
>3–6	69.6 (64.1–76.0)	21.7 (19.6–24.6)	82 (78–87)	9.2 (7.2–13.2)	1.2 (0.9–1.7)	97 (73–136)	51 (44–61)	64 (60–70)	105 (100–112)
>1–3	70.5 (65.5–77.8)	22.0 (20.1–25.1)	85 (77–91)	9.6 (6.4–13.4)	1.2 (0.8–1.7)	85 (63–151)	51 (42–58)	67 (61–71)	106 (100–115)
0–1	75.1 (69.9–82.0)	23.2 (20.9–26.7)	84 (79–90)	11.3 (7.5–17.3)	1.5 (0.9–2.2)	90 (63–138)	48 (42–58)	66 (60–74)	110 (100–119)
Time of television and video games, (hours)^b^									
<2	69.5 (63.2–75.8)**	21.3 (18.9–24.0)**	83 (77–88)	8.8 (6.1–12.7)	1.1 (0.8–1.6)	85 (65–132)*	51 (44–60)	65 (60–70)	105 (100–115)
2–4	70.1 (64.6–77.0)	21.8 (19.7–24.9)	84 (79–90)	9.4 (6.9–13.5)	1.2 (0.9–1.7)	93 (67–128)	51 (44–60)	65 (60–70)	106 (100–114)
>4	72.5 (67.4–78.4)	22.8 (20.2–25.3)	83 (79–89)	9.9 (6.9–15.2)	1.2 (0.9–2.0)	100 (73–141)	48 (40–58)	64 (60–70)	105 (100–114)
Physical activity^b^									
High	70.9 (64.5–77.7)	21.6 (19.3–24.6)*	83 (78–89)	7.9 (5.5–11.8)**	1.0 (0.7–1.5)**	84 (60–126)**	52 (44–60)**	68 (60–71)*	108 (100–113)
Moderate	70.2 (64.3–76.6)	22.0 (19.7–24.9)	84 (78–89)	9.9 (7.1–13.8)	1.2 (0.9–1.7)	98 (73–141)	51 (43–59)	64 (60–70)	104 (100–115)
Low	70.9 (65.6–77.4)	22.4 (20.0–25.3)	84 (79–90)	10.6 (7.7–15.5)	1.4 (1.0–1.9)	97 (71–137)	48 (42–57)	64 (60–70)	105 (100–114)
Percent of calories from simple carbohydrate									
≤10	70.4 (64.6–76.4)	21.7 (19.9–24.6)	83 (78–89)	8.8 (6.3–12.8)*	1.1 (0.8–1.6)*	84 (60–125)**	53 (44–61)**	64 (60–70)	105 (100–114)
>10	70.5 (64.8–77.5)	22.0 (19.5–25.0)	84 (78–90)	9.8 (6.9–14.1)	1.2 (0.9–1.8)	96 (69–141)	49 (42–58)	64 (60–70)	105 (100–114)
Consumption of fruits daily									
Yes	69.2 (63.1–75.6)**	21.5 (19.1–23.9)**	82 (77–88)**	8.8 (6.0–12.2)**	1.1 (0.8–1.55)**	89 (65–128)	51 (43–60)	64 (60–70)	103 (100–112)*
No	71.0 (65.6–77.9)	22.3 (20.0–25.3)	84 (79–90)	9.8 (6.9–14.7)	1.2 (0.9–1.8)	94 (68–137)	50 (42–59)	64 (60–70)	106 (100–115)

Data presented in median and interquartile range. *BMI* body mass index, *WC* waist circumference, *TG* triglycerides, *HDL* high density lipoprotein, *DBP* diastolic blood pressure, *SBP* systolic pressure arterial. Significant differences between groups: *P <0.05, **P <0.01 (^a^U de Mann-Whitney; ^b^ Kruskal-Wallis)

### Estimation of ancestry and its effects in relation to each of the cardio-metabolic parameters

**Fig. 1:**
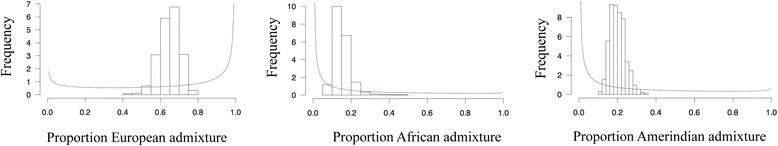
Dirichlet distribution of the ancestral components of the study population. The figure shows the Dirichlet distribution of the ancestral components of the study population using 40 ancestral informative markers (AIMs)

The range of European ancestry was 41–82 %; African, 4–48 %; and Amerindian, 10–35 %. The average ancestral percentages of the 853 young people were 66.6 ± 5.7 % European, 14.0 ± 4.7 % African, and 19.4 ± 4.1 % Amerindian. The individual admixtures are shown in Additional file 1: Table S4 and Figure S1. Dirichlet distributions of the proportions of European, African, and Amerindian ancestral components in the study population are shown in Fig. 1. Median differences and logistic regression model analyses were performed after recoding each of the cardio-metabolic parameters to convert them into binary variables. Our bivariate analyses revealed that the subjects with high TGs had a higher percentage of the Amerindian ancestry (*P* = 0.001) and less European ancestry (*P* = 0.007) than those with normal values. The subjects who had a high SBP had a lower European ancestry component (*P* = 0.002) and a higher African component (*P* = 0.013) in comparison with those who had normal SBP values. Subjects who had high insulin levels and HOMA-IR measurements had a higher percentage of African ancestry (*P* = 0.029 and *P* = 0.036, respectively) than those who had normal values in these parameters (Table 2). The values for BMI, WC, glucose levels, HDL and DBP did not show significant differences in relation to any of the ancestries.

**Table 2:** Comparison of medians of European, African and Amerindian ancestry according to components of metabolic syndrome

			European	African	Amerindian
		*n* (%)	Median (IQR)	Median (IQR)	Median (IQR)
Overall		853 (100)	0.67 (0.63–0.71)	0.13 (0.11–0.16)	0.19 (0.16–0.22)
WC	Adequate (p90^th^)	818 (96)	0.67 (0.63–0.71)	0.13 (0.11–0.16)	0.19 (0.16–0.22)
	High (>p90^th^)	35 (4)	0.67 (0.62–0.70)	0.13 (0.10–0.17)	0.19 (0.17–0.23)
BMI	Adequate (≤ p85^th^)	418 (49)	0.67 (0.63–0.71)	0.13 (0.11–0.16)	0.19 (0.16–0.22)
	Overweight (>p85^th^)	435 (51)	0.67 (0.63–0.71)	0.13 (0.11–0.16)	0.19 (0.16–0.22)
Fasting Glucose	Adequate (<100 mg/dL)	829 (97)	0.67 (0.63–0.71)	0.13 (0.11–0.16)	0.19 (0.16–0.22)
	High (≥100 mg/dL)	24 (3)	0.68 (0.64–0.71)	0.15 (0.12–0.17)	0.19 (0.15–0.20)
Fasting Insulin	Adequate (≤23)	799 (94)	0.67 (0.63–0.71)	0.13 (0.11–0.16)*	0.19 (0.16–0.22)
	High (>23,1)	54 (6)	0,66 (0,63–0,70)	0.15 (0.12–0.17)	0.19 (0.16–0.22)
HOMA score	Adequate (≤ 3.1)	808 (95)	0.67 (0.63–0.71)	0.13 (0.11–0.16)*	0.19 (0.16–0.22)
	High (>3.1)	45 (5)	0.66 (0.63–0.70)	0.15 (0.12–0.17)	0.19 (0.16–0.22)
TG	Adequate (< 100 mg/dL)	537 (63)	0.68 (0.63–0.72)**	0.13 (0.11–0.16)	0.19 (0.16–0.22)**
	High (≥110 mg/dL)	316 (37)	0.66 (0.63–0.70)	0.13 (0.11–0.16)	0.20 (0.17–0.23)
HDL	Adequate (> 40 mg/dL)	689 (81)	0.67 (0.63–0.71)	0.13 (0.11–0.16)	0.19 (0.16–0.22)
	Low (≤40 mg/dL)	164 (19)	0.67 (0.63–0.70)	0.13 (0.11–0.17)	0.19 (0.17–0.22)
DBP	Adequate (<p90^th^)	786 (92)	0.67 (0.63–0.71)	0.13 (0.11–0.16)	0.19 (0.16–0.22)
	High (≥p90^th^)	67 (8)	0.67 (0.62–0.70)	0.13 (0.11–0.16)	0.20 (0.18–0.23)
SBP	Adequate (*p* <90^th^)	811 (95)	0.67 (0.63–0.71)**	0.13 (0.11–0.16)*	0.19 (0.16–0.22)
	High (≥p90^th^)	42 (5)	0.65 (0.61–0.68)	0.15 (0.12–0.18)	0.19 (0.17–0.23)

Data presented in median and interquartile range. *BMI* body mass index, *WC* waist circumference, *TG* triglycerides, *HDL* high density lipoprotein, *DBP* diastolic blood pressure, *SBP* systolic pressure arterial, *IQR* interquartile range. Significant differences between groups: U de Mann-Whitney test *P <0.05, **P <0.01

The logistic regression model included ancestry as the principal independent variable and was adjusted for age, sex, pubertal maturation, socioeconomic stratum, physical activity, percent of calories from simple carbohydrates, daily consumption of fruits and BMI. Even though some of the covariates were not significantly associated with the outcome variables in Table 1, some of them were included, as they have been previously reported to be important factors. Amerindian ancestry was associated with an increased risk for elevated TGs for each percentage unit increase in Amerindian ancestry; specifically, the probability of having high TGs increases by 6 % (OR = 1.06, 98.3 % IC = 1.01–1.11, *P* = 0.002). After the adjustments described above, no effect of the European ancestry was found on TGs levels, but a protective effect was found for high SBP; for each percentage unit increase in European ancestry, the probability of having high SBP decreased by 7 % (OR = 0.93, 98.3 % IC = 0.87–0.99, *P* = 0.008). However, an African ancestry was positively associated with a risk for high SBP; for each percentage unit increase in African ancestry, the probability of having high SBP increased by 6 % (OR = 1.07, 98.3 % IC = 1.01–1.14, *P* = 0.008) (Table 3).

**Table 3:** Multiple logistic regression results for the association of genetic ancestry with components of metabolic syndrome

Phenotype	Ancestry	OR (CI-95 %)^a^	*P-Value* ^*a*^	OR (CI-95 %)^b^	*P-Value* ^*b*^	OR (CI-98.3 %)^c^
BMI	European	1.01 (0.99–1.04)	0.314	1.02 (0.99–1.04)	0.207	1.01 (0.99–1.05)
	African	0.99 (0.96–1.02)	0.556	0.98 (0.95–1.01)	0.317	0.98 (0.95–1.02)
	Amerindian	1.35 (0.95–1.02)	0.426	0.99 (0.95–1.02)	0.511	0.99 (0.95–1.03)
WC	European	1.00 (0.94–1.06)	0.963	1.00 (0.94–1.07)	0.880	1.00 (0.93–1.09)
	African	1.00 (0.93–1.07)	0.954	1.00 (0.93–1.07)	0.946	1.00 (0.91–1.09)
	Amerindian	0.99 (0.91–1.08)	0.870	0.99 (0.90–1.08)	0.789	0.99 (0.89–1.10)
Fasting glucose	European	1.00 (0.93–1.08)	0.938	1.03 (0.96–1.11)	0.441	1.03 (0.94–1.13)
	African	1.04 (0.97–1.12)	0.217	1.02 (0.95–1.10)	0.532	1.02 (0.94–1.11)
	Amerindian	0.92 (0.82–1.02)	0.112	0.90 (0.81–1.00)	0.062	0.90 (0.79–1.03)
Fasting insulin	European	0.98 (0.94–1.03)	0.484	0.97 (0.92–1.03)	0.367	0.97 (0.91–1.04)
	African	1.03 (0.98–1.08)	0.234	1.05 (0.99–1.11)	0.119	1.05 (0.97–1.13)
	Amerindian	0.99 (0.92–1.05)	0.671	0.98 (0.91–1.06)	0.599	0.98 (0.89–1.07)
HOMA score	European	0.98 (0.93–1.03)	0.340	0.97 (0.91–1.03)	0.299	0.97 (0.90–1.04)
	African	1.03 (0.98–1.09)	0.201	1.05 (0.99–1.12)	0.109	1.05 (0.97–1.14)
	Amerindian	0.99 (0.92–1.07)	0.869	0.98 (0.91–1.07)	0.698	0.98 (0.89–1.09)
TG	European	0.98 (0.95–1.00)	**0.049**	0.98 (0.95–1.01)	0.120	0.98 (0.95–1.01)
	African	0.99 (0.96–1.02)	0.661	0.98 (0.95–1.02)	0.369	0.98 (0.95–1.02
	Amerindian	1.06 (1.02–1.09)	**0.002**	1.06 (1.02–1.10)	**0.002**	1.06 (1.01–1.11)
HDL	European	1.00 (0.97–1.03)	0.890	1.00 (0.96–1.03)	0.843	1.00 (0.96–1.04)
	African	1.00 (0.96–1.03)	0.905	1.00 (0.96–1.04)	0.956	1.00 (0.95–1.05)
	Amerindian	1.01 (0.97–1.05)	0.610	1.01 (0.97–1.06)	0.573	1.01 (0.96–1.07)
SBP	European	0.93 (0.88–0.97)	**0.003**	0.93 (0.88–0.99)	**0.008**	0.93 (0.87–0.99)
	African	1.08 (1.03–1.13)	**0.002**	1.07 (1.02–1.13)	**0.008**	1.07 (1.01–1.14)
	Amerindian	1.01 (0.94–1.09)	0.706	1.01 (0.94–1.09)	0.728	1.01 (0.92–1.11)
DBP	European	0.98 (0.94–1.02)	0.399	0.98 (0.94–1.03)	0.433	0.98 (0.93–1.04)
	African	0.98 (0.93–1.04)	0.538	0.98 (0.92–1.04)	0.504	0.98 (0.91–1.05)
	Amerindian	1.05 (0.99–1.12)	0.078	1.06 (0.99–1.12)	0.078	1.06 (0.98–1.14)

^a^Univariate analysis

^b^All models have been adjusted for gender, age, pubertal maturation, socioeconomic stratum, physical activity, percent of calories from simple carbohydrate and consumption of fruits daily. The models for WC, fasting glucose, fasting insulin, HOMA score, TG, HDL, DBP and SBP have also been adjusted for BMI. The significant p values* (P< *0.05) are given in bold. The comparison was statistically significant according to the prespecified Bonferroni-corrected threshold of *P* <0.0167, but not for the global correction *P* <0.0018

^c^ 98.3 % Confidence Intervals (CI) are presented to be consistent with a Bonferroni correction

## Discussion

This study evaluated the association of ancestry with the following cardio-metabolic risk factors in a young population that was the product of ancestral mixture: WC, BMI, glucose, insulin, HOMA-IR, TGs, HDL, DBP and SBP. This analysis was based on 40 AIMs whose deltas (δs) among the Amerindian, European and African populations indicate that they are good discriminators (Additional file 1: Table S1). The average and individual results obtained for the ancestries of the sample population match the data obtained by other researchers in the same population using different types and numbers of AIMs [6, 8, 24]. We must note, in interpreting the following results, that we set two threshold significance as *p* <0.0167 (three ancestry), and *p* <0.0018 (three ancestries * nine cardio-metabolic parameters) to provide a conservative Bonferroni correction for multiple testing, however it may be unnecessarily rigorous when there is a priori evidence of the associations being tested. This study, using a multivariate analysis, showed an association positive between African ancestral components and SBP (Table 3), which has been widely reported in genetic studies of blood pressure (BP) conducted in African populations and in populations that are the product of genetic admixture with African populations, such as Latin American populations, including one study on an adult population from the same region where the present study was conducted [25–27]. However, despite the consistently demonstrated effect of an African ancestry component on blood pressure, other studies in the United States have found no differences in the blood pressure of children with different ethnic origins [28]. In this study, the inverse relationship found between SBP and a European ancestral component in young people from 10 to 18 years of age agrees with the findings in adults.

TG levels were positively associated with an Amerindian ancestral component, which agrees with some studies conducted on adult populations where it has been reported that Amerindian women have higher TGs levels than African American (AA) or European-American (EA) women. Additionally, the prevalence of dyslipidemia (low HDL, high LDL or high TGs) is greater in Amerindian than in European populations [29]. In 2014, Ko et al. identified eight gene variants associated with dyslipidemias. In inferring the ancestral state, they found that seven of these variants were of an Amerindian ancestry [30].

With regard to the HOMA-IR and insulin measurements, a significant difference was found when a comparison of medians was carried out with the African ancestral component. Although there was no significant difference shown by the logistic regression, it is useful to discuss these results because several studies have associated insulin resistance with African ancestry [31, 32]. Insulin and HOMA-IR are measurements related to T2D, and other studies have found a high prevalence of this disease in some AA populations [33, 34]. In addition, in a study carried out on adults from the same region from which the subjects of this study come, a significant association was found between the African ancestral component and the risk of T2D. This association reinforces the importance of considering the hypothesis of the “thrifty genotype” that has been described for North American populations, such as the Pima Indians [34], in whom there is a very high prevalence of T2D. This prevalence is believed to be related to the presence of genes variants adapted to the dispersion of these populations across the Bering Strait [34]. Other studies in admixed populations found no association of the Amerindian component with fasting blood glucose levels or with HOMA-IR [25, 35]. In two studies, one conducted in Medellín [17] and the other in Mexico [18], where a positive association of T2D with the Amerindian genetic component was reported, the authors suggest that this association could be explained because the low socioeconomic stratum has a higher Amerindian genetic component than that of the other strata, and the lifestyle and environmental factors of this stratum favor the development of T2D. The etiology of this complex trait has a high epigenetic component; it has been demonstrated that early exposure to hyperglycemia predisposes individuals to changes in their chromatin and gene expression, which can be transmitted from one generation to another (metabolic memory), and this might be what was observed in the African component in the present study, which showed an association with the insulin resistance parameters [36].

No associations of any of the three ancestral components were detected with BMI, WC, HDL and glucose level despite the high heritabilities found for these traits [37]. Additionally, a relationship to ancestry has been demonstrated in several studies on populations in the United States, in which it has been found that adiposity is higher in EA and Hispanic-American (HA) children than in AA children [38, 39]. It has been reported that WC in Mexican-American children is greater than in EA and AA children [14]. Since 2000, higher levels of HDL have been reported in both children and adults in AAs and HAs than in EAs [40]. Based on this evidence, it was expected that there would be some degree of association between the genetic components of the population and these character traits. However, the negative results presented here could demonstrate that the association between these traits and the population’s genetic components is different, not only with respect to age but also with respect to the ancestral genetic architecture, which depends on the demographic history of the population. With respect to studies conducted on associations with the complete genome, evidence has shown the existence of genes associated with BMI and WC in both adults and children, while other associations are present only in adults or only in children [41]. This shows the complexity of the inheritance of these traits. For example, a recent study found an inverse relationship between 31 loci associated with the risk of obesity, as defined by BMI, and changes in weight in relation to age [42].

The results of this study seem to agree with the ideas of Joshi et al. [42], which suggest directional dominance. They assessed whether homozygosis in certain regions of the genome had any directional relationship with quantitative traits such as height, weight, cognitive measures and cardio-metabolic parameters, and they found that only height and cognition clearly presented any directional dominance in continental populations. However, the cardio-metabolic measurements (like those evaluated in the present study) did not present it, and it was shown to have a strong variation with age [43].

## Conclusion

Here we have shown that although the effect of ancestry was modest, it was significant, and based on these findings, we suggest that an Amerindian ancestry may act as a risk factor for high triglycerides, while an African ancestral component confers a risk for high systolic blood pressure. A European ancestry serves as a protective factor for this condition in the young people of the population studied here. In addition, our results show that ancestry is not associated with WC, BMI, HDL or glucose levels. It is necessary to extend this study to include more AIMs and a greater sample size in the hopes of replicating these results and, thus, enabling the use of predictions based on ancestral components, either as risk or protection factors, to determine prevention strategies or to identify candidate genes for cardio-metabolic disorders in children.

## Abbreviations

MetS, metabolic syndrome; DNA, deoxyribonucleic acid; AIMs, ancestry-informative markers; LA, Latin America; DANE, National Administrative Department of Statistics; WC, waist circumference; TGs, triglycerides, HDL, high-density lipoprotein; DBP, diastolic blood pressure; SBP, systolic blood pressure; T2D, type 2 diabetes mellitus; BMI, body mass index; HOMA, homeostasis model assessment; IR, insulin resistance; PCR-RFLP, polymerase chain reaction-restriction fragment length polymorphism); IN/DEL, insertion-deletion; OR, odds ratio; CIs, confidence intervals; EAs, European-Americans; HAs, Hispanic-Americans; AAs, African Americans

## Additional file

Additional file 1: Table S1. Prevalence of diseases/trait cardio-metabolic, Age standardized estimate by region. **Table S2.** Ancestral informative Markers (AIMs), allelic frequencies and delta (δ) of frequencies in the three ancestral populations (European, African and Amerindian). **Table S3.** Sequence of primers and amplification parameters of the ancestry informative markers (AIMs). **Table S4.** Individual ancestral composition. **Figure S1.** Individual ancestral composition. (DOCX 155 kb)
