# Supplementary material for: Cardio-metabolic parameters are associated with genetic admixture estimates in a pediatric population from Colombia
Source: BMC Genet. 2016 Jun 27;17:93. doi: 10.1186/s12863-016-0402-5 (PMC4924275; doi:10.1186/s12863-016-0402-5)
Supplement: Additional file 1: Table S1. — Prevalence of diseases/trait cardio-metabolic, Age standardized estimate by region. Table S2. Ancestral informative Markers (AIMs), allelic frequencies and delta (δ) of frequencies in the three ancestral populations (European, African and Amerindian). Table S3. Sequence of primers and amplification parameters of the ancestry informative markers (AIMs). Table S4. Individual ancestral composition. Figure S1. Individual ancestral composition. (DOCX 155 kb) [file 12863_2016_402_MOESM1_ESM.docx]

**Table S1. Prevalence of diseases/trait cardio-metabolic, Age standardized estimate by region**

|  | AFR | | EUR | ME/N.AFR | N.AME/CAR | SCA | reference | |  |
| --- | --- | --- | --- | --- | --- | --- | --- | --- | --- |
|  | | Ages ≥20 -79 years | | | | | |  | |
|  | Percentage (%) | | | | | | | |  |
| Diabetes* | | | | | | | | |  |
|  | 3.8 | | 7.3 | 9.6 | 10.7 | 11.5 | [1] | |  |
| Impaired glucose tolerance* | | | | | | | | |  |
|  | 9.1 | | 4.1 | 8.0 | 8.6 | 13.9 |  | |  |
|  |  | |  |  |  |  |  | |  |
|  | AFR | | EUR |  | AMR | |  | |  |
|  | Ages ≥18+ years | | | | | |  | |  |
| Overweight, BMI ≥ 25kg/m^2^ * | | | | | | | | |  |
|  | 30.8 | | 58.6 | - | 61.0 | - | [2] | |  |
| Obesity, BMI ≥ 30kg/m^2^ * | | | | | | | | |  |
|  | 10.4 | | 23.0 | - | 26.8 | - | [2] | |  |
| Raised blood pressure,  (SBP ≥ 140 or DBP ≥ 90)* | | | | | | | | |  |
|  | 29.6 | | 23.3 | - | 18.2 | - | [2] | |  |
| Raised total cholesterol  (≥5.0 mmol/L)* | | | | | | | | |  |
|  | 22.6 | | 54.0 | - | 48.0 | - | [2] | |  |

AFR: Africa , EUR: Europe, ME/NAFR: Middle East and North Africa, N.AME/CAR: North America and Caribbean, SCA: South and Central America, AMR: Americas, BMI: body mass index, SBP: sistolic blood pressure, DBP: diastolic blood pressure

References

1. International Diabetes Federation. IDF Diabetes Atlas, 7 ed. Brussels, Belgium: International Diabetes Federation, 2015.
2. World Health Organization. Global Health Observatory Data Repository. Noncommunicable disease:Risk factors by WHO region. Available from: <http://www.who.int/gho/ncd/risk_factors/en/> [cited 24 January 2016].

**Table S2. Ancestral informative Markers (AIMs), allelic frequencies and delta (δ) of frequencies in the three ancestral populations (European, African and Amerindian)**

| AIM | Chr | Phys. Loc.  (bp) | Freq  Eu | Freq  Am | Freq  Af | δ  (Af-Eu) | δ  (Af-Am) | δ  (Eu-Am) |
| --- | --- | --- | --- | --- | --- | --- | --- | --- |
| *MID1752* | 1 | 55048581 | 0.290 | 0.920 | 0.560 | 0.270 | 0.360 | **0.630** |
| *FYNULL* | 1 | 164822103 | 0.998 | 1.000 | 0.001 | **0.997** | **0.999** | 0.002 |
| *AT3* | 1 | 170618399 | 0.282 | 0.061 | 0.858 | **0.576** | **0.797** | 0.221 |
| *MID1386* | 1 | 244094748 | 0.730 | 0.070 | 0.770 | 0.040 | **0.700** | **0.660** |
| *NBC4* | 2 | 74296877 | 0.683 | 0.947 | 0.513 | 0.170 | **0.430** | 0.264 |
| *MID921* | 3 | 708993 | 0.090 | 0.060 | 0.690 | **0.600** | **0.630** | 0.030 |
| *Ya5ACA1100* | 3 | 156131069 | 1.000 | 1.000 | 0.450 | **0.550** | **0.550** | 0.000 |
| *D1* | 3 | 179593741 | 0.011 | 0.500 | 0.256 | 0.245 | 0.244 | **0.489** |
| *Ya5ACA1184* | 4 | 41782815 | 0.688 | 0.947 | 0.158 | **0.530** | **0.789** | 0.259 |
| *GC-1F* | 4 | 72983369 | 0.156 | 0.339 | 0.853 | **0.697** | **0.514** | 0.183 |
| *MID1586* | 4 | 88316243 | 0.420 | 0.260 | 0.960 | **0.540** | **0.700** | 0.160 |
| *MID52* | 4 | 201758344 | 0.840 | 0.140 | 0.740 | 0.100 | **0.600** | **0.700** |
| *Ya5ACA1153* | 4 | 181786436 | 0.269 | 0.833 | 0.306 | 0.037 | **0.527** | **0.564** |
| *MID817* | 5 | 11400042 | 0.650 | 0.130 | 0.960 | 0.310 | **0.830** | **0.520** |
| *MID1039* | 5 | 34009694 | 0.270 | 0.830 | 0.980 | **0.710** | 0.150 | **0.560** |
| *MID1358* | 5 | 35655730 | 0.060 | 0.040 | 0.800 | **0.740** | **0.760** | 0.020 |
| *MID856* | 5 | 65320596 | 0.150 | 0.690 | 0.660 | **0.510** | 0.030 | **0.540** |
| *MID944* | 5 | 92340215 | 0.390 | 0.950 | 0.890 | **0.500** | 0.060 | **0.560** |
| *pAlu6-17534722* | 6 | 17580264 | 0.750 | 0.375 | 0.125 | **0.625** | 0.250 | 0.375 |
| *MID108* | 6 | 32808464 | 0.320 | 0.040 | 0.580 | 0.260 | **0.540** | 0.280 |
| *MID104* | 6 | 32810633 | 0.350 | 0.110 | 0.560 | 0,210 | **0.450** | 0.240 |
| *MID2062* | 6 | 40194876 | 0.290 | 0.930 | 0.410 | 0.120 | **0.520** | **0.640** |
| *Ya5ACA1702* | 7 | 42614187 | 0.800 | 1.000 | 0.500 | 0.300 | **0.500** | 0.200 |
| *Ya5ACA1611* | 7 | 113740756 | 0.611 | 1.000 | 0.211 | 0.400 | **0.789** | **0.389** |
| *LPL* | 8 | 19859469 | 0.492 | 0.442 | 0.971 | **0.479** | **0.529** | 0.050 |
| *Ya5aca1861* | 9 | 26502940 | 0.417 | 0.889 | 0.875 | **0.458** | 0.014 | **0.472** |
| *MID1780* | 11 | 35103909 | 0.230 | 0.690 | 0.740 | **0.510** | 0.050 | **0.460** |
| *B65* | 11 | 89572171 | 0.489 | 0.270 | 0.830 | 0.341 | **0.560** | 0.219 |
| *DRD2* | 11 | 112796798 | 0.670 | 0.045 | 0.135 | **0.535** | 0.090 | **0.625** |
| *APOA* | 11 | 123600000 | 0.925 | 0.977 | 0.420 | **0.505** | **0.557** | 0.052 |
| *MID1723* | 12 | 79547087 | 0.180 | 0.150 | 0.900 | **0.720** | **0.750** | 0.030 |
| *RB2300* | 13 | 47776293 | 0.315 | 0.175 | 0.926 | **0.611** | **0.751** | 0.140 |
| *MID2264* | 13 | 62676757 | 0.300 | 1.000 | 1.000 | **0.700** | 0.000 | **0.700** |
| *MID2269* | 13 | 85138935 | 0.400 | 0.900 | 0.900 | **0.500** | 0.000 | **0.500** |
| *OCA2* | 15 | 25909368 | 0.746 | 0.488 | 0.115 | **0.631** | 0.373 | 0.258 |
| *MID818* | 16 | 2140623 | 0.780 | 0.980 | 0.090 | **0.690** | **0.890** | 0.200 |
| *PV92* | 16 | 87700000 | 0.152 | 0.792 | 0.225 | 0.073 | **0.567** | **0.640** |
| *Ya5_435* | 17 | 64599263 | 0.400 | 1.000 | 0.200 | 0.200 | **0.800** | **0.800** |
| *SB19.3* | 19 | 27200000 | 0.903 | 0.645 | 0.415 | **0.488** | 0.230 | 0.258 |
| *MID154* | 20 | 32140667 | 0.250 | 0.140 | 0.820 | **0.570** | **0.680** | 0.110 |

Chr: Chromosome; Phys. Loc: physical location; Freq: Allele frequency; Eu: European; Am: Amerindian; Af: African; δ: Differences among the continental populations. Equals to differences between pairs of continental populations. δ >0.40 is shown in bold.

**Table S3. Sequence of primers and amplification parameters of the ancestry informative markers (AIMs)**

| **LOCUS** | **Chr.** | **Variant type** | **Reverse Primer** | **Forward Primer** | **Tm** | **Fluorochrome or enzyme** |
| --- | --- | --- | --- | --- | --- | --- |
| *MID1752* | 1 | I/D | AGATTGACATTCCTTCCACA | TGTTTGTACCTTCCAAGTCTCT | 59.5 | NA |
| *FYNULL* | 1 | T/C | GGCATAGGGATAAGGGACT | AGGCTTGTGCAGGCAGTG | 58.5 | RsaI |
| *AT3* | 1 | I/D | GAGATAGTGTGATCTGAGGC | CCACAGGTGTAACATTGTGT | 57.0 | NA |
| *MID1386* | 1 | I/D | GGATTGATGTTCCAAGTCAG | AGAACACATGAGCTGAGAGG | 57.5 | NA |
| *NBC4* | 2 | I/D | GCTTTGGAAGTAGGCAGGTTAC | CATTCCACCCTGTCAGCATT | 60.0 | NA |
| *MID921* | 3 | I/D | GAGGTTCCCTCTGTATGTAGC | GAGGTTCCCTCTGTATGTAGC | 65.0 | TET |
| *Ya5ACA1100* | 3 | I/D | GCCTGGGCAATAATTTTCAA | GCATCCTACAAAGCCATT | 56.2 | NA |
| *D1* | 3 | I/D | TTTCTGCTATGCTCTTCCCTCTC | TGCTGATGCCCAGGGTTAGTAAA | 60.0 | NA |
| *Ya5ACA1184* | 4 | I/D | CCCAGGTGATTCATTCCATC | TGGCTCTAATGACCAAAAGGA | 56.2 | NA |
| *GCH* | 4 | T/G | GGAGGTGAGTTTATGGAACAGC | AGATCTGAAATGGCTATTATTTTGC | 57.5 | Haelll |
| *MID1586* | 4 | I/D | CCACAGAAGGCTCAGTCTTA | GTTATGTGGGCAGTGTTTTC | 55.0 | FAM |
| *MID52* | 4 | I/D | GTTCCAGTTGTTGGTGTGAC | GCACAGGTGTTTTAGAGGC | 55.5 | NA |
| *Ya5ACA1153* | 4 | I/D | CCTGGGTTGTCCTTCTGTGT | GGCAGGAGTTAGCCAGGTAG | 62.9 | NA |
| *MID817* | 5 | I/D | CCTACATCCAACAGAAGGTG | ATTACCGGAACACATTCTGA | 57.5 | NA |
| *MID1039* | 5 | I/D | GCTTTCTTCATTCCTTCACA | CGTTTCATCTCTTTGGGTTA | 57.5 | NA |
| *MID1358* | 5 | I/D | AGACGCCAGGAATTTTCTAT | GTTTTGGGAATTTAGGTTTTG | 57.5 | NA |
| *MID856* | 5 | I/D | TATTGTGCTCATTTTCTGGG | AACATGGGAACTGCTCATTA | 57.5 | NA |
| *MID944* | 5 | I/D | GTAAGCAGCCTGGATTACAA | TCAGTAAAAGGGTTTCCTTGT | 57.5 | NA |
| *pAlu6-17534722* | 6 | I/D | GTGTTGGTAGTGAAGAGAGCCAAC | TACCTCGATAGTCTCACTTC | 56.0 | NA |
| *MID108* | 6 | I/D | GCATCTGTTGCCATTGTT | CTCCTCCTCATCCAAAAATT | 52.5 | TET |
| *MID104* | 6 | I/D | CAGAGGGTCTAGAGCAAAATT | CCTTAGCTCAGTATGCTCCA | 56.0 | NA |
| *MID2062* | 6 | I/D | GAAGCCAGAACAATGAAAGA | GGCCTGCATGATAAATAGAA | 57.5 | NA |
| *Ya5ACA1702* | 7 | I/D | TGGATAAAGCTGCAACACCA | TCCCCCATCTCTTTTTCCTT | 61.0 | NA |
| *Ya5ACA1611* | 7 | I/D | TCCTAAACATAATACGTACAGGTGA | TTTTGGTAAAGATGCCACAGAA | 62.0 | NA |
| *LPL* | 8 | C/T | TTATGCTGCTTTAGACTCTTGTC | AGGCTTCACTCATCCGTGCCTCC | 57.5 | PvuII |
| *Ya5ACA1861* | 9 | I/D | GGCTCGGTTGGTTGTTTTAG | TATTTCCACCAAGAGGGAGC | 64.0 | NA |
| *MID1780* | 11 | I/D | ACACTTGCAGAGAGCTTTGT | TGACTTCAGTGTCTGCTGAA | 57.5 | TET |
| *B65* | 11 | I/D | TGAGTTATTTCCCCTCTCCG | CTAAAAGGGACACCAGCCCT | 64.8 | NA |
| *DRD2* | 11 | T/C | AAAACTAGGGAGGGTCAGAG | CCTCTGAGGCTTACTGTCTG | 56.0 | TaqI |
| *APOA* | 11 | I/D | AGTCTTCGATGACAGCGTATACAGA | AAGTGCTGTAGGCCATTTAGATTAG | 50.0 | NA |
| *MID1723* | 12 | I/D | GAGCAAAAGTGTAATTTCCCT | CTTCAAACTATGGTCTTCAAAAA | 55.0 | HEX |
| *RB2300* | 13 | G/A | CTGCAGACGCTCCGCCGT | CAGGACAGCGGCCCGGAG | 60.0 | BamHI |
| *MID2264* | 13 | I/D | CCACTCACATTCCAATTTCA | TGTGAGGTAAGGACCCAATT | 55.0 | FAM |
| *MID2269* | 13 | I/D | ACCAGAGTGGCTACTTTTGG | TTTCTCCACTGCGTTCAGTA | 55.0 | FAM |
| *OCA2* | 15 | A/G | ACCTCTAGCATGGTTCTTGGGC | CTTTCGTGTGTGCTAACTCC | 65.0 | HaeIII |
| *MID818* | 16 | I/D | ACTTCAGTCGTCACTCCATC | TAGAGCCAGTTAGAGGGAGG | 57.5 | NA |
| *PV92* | 16 | I/D | GAAAGGCAAGCTACCAGAAGCCCCAA | GGATCTCAGGGTGGGTGGCAATGCT | 65.0 | NA |
| *Ya5_435* | 17 | I/D | AAAAGGTAATCCCTCTATCCTCTTG | CTGGCGACTAAGGTGAAAGC | 63.0 | NA |
| *SB19,3* | 19 | I/D | AAGCACAATTGGTTATTTTCTGAC | TCTAGCCCCAGATTTATGGTAACTG | 60.0 | NA |
| *MID154* | 20 | I/D | AACAGGCAATCCTCCTAAGT | GGCTCTGACTGAGAAACTGA | 57.5 | NA |

Chr: Chromosome; Tm: annealing temperature

**Table S4. Individual ancestral composition**

| ID | Individual Ancestral Composition * | | |
| --- | --- | --- | --- |
|  | Europen | African | Amerindian |
| 1001 | 0.64 | 0.14 | 0.22 |
| 1003 | 0.67 | 0.17 | 0.17 |
| 1005 | 0.61 | 0.17 | 0.22 |
| 1006 | 0.7 | 0.14 | 0.16 |
| 1007 | 0.65 | 0.16 | 0.19 |
| 1009 | 0.71 | 0.13 | 0.17 |
| 1010 | 0.58 | 0.23 | 0.2 |
| 1012 | 0.64 | 0.17 | 0.2 |
| 1013 | 0.66 | 0.14 | 0.21 |
| 1014 | 0.54 | 0.18 | 0.27 |
| 1016 | 0.66 | 0.2 | 0.15 |
| 1019 | 0.74 | 0.13 | 0.13 |
| 1020 | 0.71 | 0.09 | 0.2 |
| 1021 | 0.64 | 0.16 | 0.2 |
| 1023 | 0.7 | 0.13 | 0.17 |
| 1025 | 0.75 | 0.1 | 0.16 |
| 1027 | 0.64 | 0.15 | 0.22 |
| 1028 | 0.62 | 0.2 | 0.18 |
| 1029 | 0.67 | 0.16 | 0.16 |
| 1031 | 0.66 | 0.13 | 0.21 |
| 1035 | 0.69 | 0.13 | 0.18 |
| 1038 | 0.63 | 0.19 | 0.19 |
| 1041 | 0.72 | 0.12 | 0.16 |
| 1044 | 0.71 | 0.17 | 0.12 |
| 1045 | 0.58 | 0.18 | 0.24 |
| 1047 | 0.68 | 0.13 | 0.19 |
| 1048 | 0.74 | 0.12 | 0.15 |
| 1049 | 0.66 | 0.18 | 0.17 |
| 1050 | 0.67 | 0.14 | 0.19 |
| 1051 | 0.68 | 0.12 | 0.2 |
| 1052 | 0.66 | 0.09 | 0.25 |
| 1053 | 0.62 | 0.14 | 0.24 |
| 1054 | 0.65 | 0.14 | 0.21 |
| 1055 | 0.62 | 0.17 | 0.21 |
| 1056 | 0.7 | 0.12 | 0.18 |
| 1057 | 0.59 | 0.18 | 0.23 |
| 1060 | 0.69 | 0.1 | 0.21 |
| 1062 | 0.76 | 0.09 | 0.14 |
| 1063 | 0.64 | 0.1 | 0.26 |
| 1064 | 0.57 | 0.14 | 0.29 |
| 1066 | 0.6 | 0.12 | 0.28 |
| 1069 | 0.76 | 0.11 | 0.14 |
| 1071 | 0.63 | 0.11 | 0.26 |
| 1072 | 0.7 | 0.09 | 0.21 |
| 1073 | 0.52 | 0.14 | 0.35 |
| 1079 | 0.73 | 0.09 | 0.18 |
| 1080 | 0.56 | 0.17 | 0.27 |
| 1081 | 0.67 | 0.1 | 0.23 |
| 1082 | 0.72 | 0.09 | 0.19 |
| 1083 | 0.52 | 0.33 | 0.15 |
| 1085 | 0.7 | 0.14 | 0.16 |
| 1088 | 0.67 | 0.12 | 0.21 |
| 1089 | 0.68 | 0.13 | 0.19 |
| 1090 | 0.69 | 0.15 | 0.17 |
| 1092 | 0.72 | 0.09 | 0.19 |
| 1094 | 0.62 | 0.14 | 0.24 |
| 1097 | 0.52 | 0.13 | 0.35 |
| 1099 | 0.67 | 0.12 | 0.21 |
| 1100 | 0.68 | 0.13 | 0.19 |
| 1101 | 0.63 | 0.15 | 0.22 |
| 1102 | 0.59 | 0.13 | 0.28 |
| 1103 | 0.6 | 0.26 | 0.15 |
| 1105 | 0.62 | 0.1 | 0.28 |
| 1106 | 0.58 | 0.1 | 0.32 |
| 1107 | 0.69 | 0.14 | 0.17 |
| 1108 | 0.72 | 0.13 | 0.15 |
| 1112 | 0.62 | 0.14 | 0.23 |
| 1113 | 0.56 | 0.22 | 0.23 |
| 1115 | 0.67 | 0.13 | 0.2 |
| 1119 | 0.66 | 0.2 | 0.14 |
| 1120 | 0.75 | 0.13 | 0.13 |
| 1121 | 0.59 | 0.15 | 0.27 |
| 1123 | 0.65 | 0.21 | 0.15 |
| 1128 | 0.66 | 0.18 | 0.16 |
| 1130 | 0.69 | 0.12 | 0.19 |
| 1134 | 0.75 | 0.12 | 0.14 |
| 1135 | 0.74 | 0.09 | 0.17 |
| 1138 | 0.7 | 0.13 | 0.18 |
| 1139 | 0.63 | 0.18 | 0.2 |
| 1141 | 0.69 | 0.16 | 0.16 |
| 1143 | 0.68 | 0.12 | 0.21 |
| 1146 | 0.6 | 0.19 | 0.21 |
| 1149 | 0.68 | 0.13 | 0.2 |
| 1150 | 0.56 | 0.26 | 0.18 |
| 1156 | 0.63 | 0.21 | 0.16 |
| 1157 | 0.65 | 0.15 | 0.2 |
| 1158 | 0.66 | 0.1 | 0.24 |
| 1161 | 0.68 | 0.1 | 0.21 |
| 1162 | 0.61 | 0.1 | 0.28 |
| 1164 | 0.63 | 0.11 | 0.26 |
| 1167 | 0.63 | 0.21 | 0.16 |
| 1170 | 0.67 | 0.1 | 0.23 |
| 1171 | 0.62 | 0.19 | 0.2 |
| 1172 | 0.46 | 0.41 | 0.12 |
| 1174 | 0.68 | 0.13 | 0.19 |
| 1175 | 0.7 | 0.14 | 0.16 |
| 1177 | 0.67 | 0.11 | 0.22 |
| 1180 | 0.61 | 0.17 | 0.22 |
| 1181 | 0.57 | 0.16 | 0.27 |
| 1182 | 0.7 | 0.11 | 0.19 |
| 1183 | 0.63 | 0.13 | 0.24 |
| 1185 | 0.67 | 0.1 | 0.22 |
| 1186 | 0.7 | 0.12 | 0.18 |
| 1187 | 0.71 | 0.14 | 0.15 |
| 1188 | 0.64 | 0.08 | 0.28 |
| 1191 | 0.68 | 0.16 | 0.16 |
| 1192 | 0.72 | 0.1 | 0.18 |
| 1193 | 0.64 | 0.09 | 0.27 |
| 1194 | 0.64 | 0.13 | 0.23 |
| 1195 | 0.66 | 0.11 | 0.23 |
| 1197 | 0.73 | 0.14 | 0.13 |
| 1200 | 0.56 | 0.21 | 0.24 |
| 1201 | 0.61 | 0.18 | 0.21 |
| 1202 | 0.61 | 0.16 | 0.23 |
| 1203 | 0.68 | 0.12 | 0.2 |
| 1204 | 0.74 | 0.1 | 0.16 |
| 1205 | 0.74 | 0.11 | 0.15 |
| 1206 | 0.76 | 0.09 | 0.14 |
| 1207 | 0.78 | 0.09 | 0.13 |
| 1208 | 0.7 | 0.11 | 0.19 |
| 1210 | 0.66 | 0.13 | 0.22 |
| 1212 | 0.62 | 0.13 | 0.25 |
| 1213 | 0.62 | 0.15 | 0.23 |
| 1215 | 0.71 | 0.12 | 0.18 |
| 1217 | 0.67 | 0.08 | 0.25 |
| 1221 | 0.7 | 0.13 | 0.17 |
| 1223 | 0.63 | 0.13 | 0.24 |
| 1224 | 0.67 | 0.13 | 0.2 |
| 1225 | 0.7 | 0.16 | 0.14 |
| 1226 | 0.67 | 0.08 | 0.24 |
| 1228 | 0.61 | 0.18 | 0.21 |
| 1230 | 0.64 | 0.14 | 0.22 |
| 1231 | 0.67 | 0.18 | 0.15 |
| 1232 | 0.67 | 0.11 | 0.22 |
| 1233 | 0.61 | 0.09 | 0.3 |
| 1234 | 0.68 | 0.13 | 0.19 |
| 1235 | 0.68 | 0.18 | 0.14 |
| 1239 | 0.67 | 0.14 | 0.2 |
| 1241 | 0.53 | 0.3 | 0.16 |
| 1242 | 0.73 | 0.12 | 0.15 |
| 1243 | 0.6 | 0.21 | 0.19 |
| 1245 | 0.72 | 0.13 | 0.15 |
| 1249 | 0.42 | 0.38 | 0.2 |
| 1252 | 0.71 | 0.09 | 0.2 |
| 1253 | 0.62 | 0.16 | 0.23 |
| 1254 | 0.6 | 0.18 | 0.22 |
| 1258 | 0.62 | 0.15 | 0.23 |
| 1259 | 0.71 | 0.1 | 0.19 |
| 1260 | 0.61 | 0.09 | 0.3 |
| 1261 | 0.69 | 0.13 | 0.18 |
| 1262 | 0.72 | 0.09 | 0.2 |
| 1263 | 0.64 | 0.13 | 0.23 |
| 1264 | 0.52 | 0.14 | 0.34 |
| 1265 | 0.67 | 0.11 | 0.22 |
| 1267 | 0.61 | 0.17 | 0.21 |
| 1269 | 0.6 | 0.19 | 0.21 |
| 1271 | 0.64 | 0.11 | 0.25 |
| 1274 | 0.74 | 0.09 | 0.17 |
| 1275 | 0.57 | 0.25 | 0.18 |
| 1276 | 0.67 | 0.15 | 0.17 |
| 1278 | 0.74 | 0.13 | 0.13 |
| 1279 | 0.62 | 0.22 | 0.16 |
| 1280 | 0.72 | 0.08 | 0.2 |
| 1281 | 0.55 | 0.19 | 0.26 |
| 1285 | 0.61 | 0.13 | 0.26 |
| 1287 | 0.68 | 0.16 | 0.16 |
| 1290 | 0.61 | 0.16 | 0.24 |
| 1294 | 0.68 | 0.13 | 0.19 |
| 1295 | 0.62 | 0.17 | 0.21 |
| 1296 | 0.67 | 0.13 | 0.21 |
| 1298 | 0.68 | 0.13 | 0.19 |
| 1299 | 0.73 | 0.11 | 0.16 |
| 1301 | 0.57 | 0.24 | 0.19 |
| 1302 | 0.69 | 0.1 | 0.2 |
| 1303 | 0.63 | 0.12 | 0.24 |
| 1306 | 0.7 | 0.09 | 0.2 |
| 1308 | 0.6 | 0.14 | 0.27 |
| 1309 | 0.65 | 0.19 | 0.17 |
| 1310 | 0.8 | 0.07 | 0.13 |
| 1311 | 0.65 | 0.11 | 0.25 |
| 1312 | 0.72 | 0.09 | 0.19 |
| 1313 | 0.53 | 0.21 | 0.27 |
| 1314 | 0.63 | 0.15 | 0.22 |
| 2001 | 0.61 | 0.16 | 0.23 |
| 2004 | 0.68 | 0.1 | 0.22 |
| 2005 | 0.67 | 0.1 | 0.22 |
| 2008 | 0.76 | 0.1 | 0.15 |
| 2011 | 0.64 | 0.21 | 0.15 |
| 2012 | 0.69 | 0.15 | 0.16 |
| 2015 | 0.72 | 0.16 | 0.13 |
| 2019 | 0.66 | 0.16 | 0.18 |
| 2020 | 0.68 | 0.15 | 0.18 |
| 2021 | 0.69 | 0.12 | 0.2 |
| 2022 | 0.57 | 0.26 | 0.17 |
| 2023 | 0.67 | 0.14 | 0.19 |
| 2024 | 0.68 | 0.14 | 0.18 |
| 2026 | 0.73 | 0.1 | 0.17 |
| 2027 | 0.67 | 0.13 | 0.2 |
| 2028 | 0.73 | 0.11 | 0.16 |
| 2029 | 0.58 | 0.18 | 0.24 |
| 2030 | 0.59 | 0.19 | 0.22 |
| 2031 | 0.63 | 0.13 | 0.24 |
| 2032 | 0.65 | 0.11 | 0.24 |
| 2033 | 0.68 | 0.11 | 0.22 |
| 2034 | 0.7 | 0.11 | 0.19 |
| 2035 | 0.59 | 0.1 | 0.31 |
| 2037 | 0.58 | 0.24 | 0.19 |
| 2038 | 0.69 | 0.12 | 0.19 |
| 2041 | 0.61 | 0.1 | 0.3 |
| 2043 | 0.67 | 0.12 | 0.21 |
| 2044 | 0.66 | 0.18 | 0.17 |
| 2048 | 0.57 | 0.14 | 0.29 |
| 2049 | 0.63 | 0.12 | 0.25 |
| 2050 | 0.66 | 0.17 | 0.18 |
| 2051 | 0.7 | 0.1 | 0.2 |
| 2053 | 0.69 | 0.14 | 0.17 |
| 2054 | 0.74 | 0.1 | 0.16 |
| 2055 | 0.54 | 0.2 | 0.26 |
| 2056 | 0.6 | 0.16 | 0.24 |
| 2057 | 0.61 | 0.16 | 0.23 |
| 2058 | 0.6 | 0.24 | 0.16 |
| 2059 | 0.62 | 0.18 | 0.21 |
| 2060 | 0.71 | 0.09 | 0.21 |
| 2061 | 0.63 | 0.09 | 0.28 |
| 2062 | 0.63 | 0.13 | 0.25 |
| 2064 | 0.54 | 0.33 | 0.13 |
| 2068 | 0.64 | 0.14 | 0.22 |
| 2069 | 0.64 | 0.16 | 0.2 |
| 2070 | 0.73 | 0.09 | 0.18 |
| 2073 | 0.68 | 0.11 | 0.21 |
| 2076 | 0.59 | 0.15 | 0.26 |
| 2081 | 0.65 | 0.13 | 0.22 |
| 2083 | 0.78 | 0.08 | 0.14 |
| 2084 | 0.68 | 0.15 | 0.17 |
| 2085 | 0.69 | 0.12 | 0.2 |
| 2087 | 0.61 | 0.16 | 0.22 |
| 2089 | 0.72 | 0.12 | 0.16 |
| 2092 | 0.74 | 0.09 | 0.17 |
| 2094 | 0.56 | 0.27 | 0.17 |
| 2096 | 0.68 | 0.18 | 0.14 |
| 2099 | 0.76 | 0.13 | 0.11 |
| 2100 | 0.69 | 0.15 | 0.16 |
| 2101 | 0.58 | 0.2 | 0.22 |
| 2102 | 0.78 | 0.09 | 0.14 |
| 2104 | 0.76 | 0.08 | 0.16 |
| 2105 | 0.58 | 0.19 | 0.23 |
| 2107 | 0.69 | 0.14 | 0.17 |
| 2109 | 0.75 | 0.11 | 0.14 |
| 2110 | 0.71 | 0.09 | 0.2 |
| 2111 | 0.75 | 0.11 | 0.14 |
| 2114 | 0.67 | 0.1 | 0.23 |
| 2115 | 0.71 | 0.15 | 0.14 |
| 2117 | 0.63 | 0.12 | 0.25 |
| 2118 | 0.72 | 0.1 | 0.18 |
| 2120 | 0.66 | 0.12 | 0.23 |
| 2122 | 0.66 | 0.12 | 0.21 |
| 2124 | 0.73 | 0.14 | 0.13 |
| 2125 | 0.64 | 0.11 | 0.25 |
| 2127 | 0.69 | 0.13 | 0.18 |
| 2129 | 0.63 | 0.1 | 0.27 |
| 2130 | 0.69 | 0.16 | 0.15 |
| 2132 | 0.53 | 0.29 | 0.18 |
| 2133 | 0.71 | 0.13 | 0.16 |
| 2134 | 0.63 | 0.17 | 0.2 |
| 2135 | 0.7 | 0.08 | 0.22 |
| 2137 | 0.7 | 0.08 | 0.22 |
| 2138 | 0.62 | 0.16 | 0.22 |
| 2140 | 0.69 | 0.13 | 0.18 |
| 2141 | 0.62 | 0.17 | 0.22 |
| 2142 | 0.7 | 0.11 | 0.2 |
| 2143 | 0.72 | 0.11 | 0.17 |
| 2144 | 0.65 | 0.11 | 0.24 |
| 2147 | 0.76 | 0.11 | 0.14 |
| 2148 | 0.65 | 0.13 | 0.22 |
| 2150 | 0.59 | 0.13 | 0.29 |
| 2151 | 0.71 | 0.14 | 0.16 |
| 2154 | 0.65 | 0.13 | 0.22 |
| 2157 | 0.59 | 0.11 | 0.3 |
| 2159 | 0.6 | 0.2 | 0.2 |
| 2160 | 0.69 | 0.16 | 0.15 |
| 2164 | 0.69 | 0.13 | 0.19 |
| 2165 | 0.65 | 0.15 | 0.2 |
| 2167 | 0.7 | 0.14 | 0.16 |
| 2169 | 0.7 | 0.1 | 0.2 |
| 3004 | 0.66 | 0.13 | 0.22 |
| 3005 | 0.68 | 0.18 | 0.14 |
| 3007 | 0.67 | 0.12 | 0.21 |
| 3008 | 0.68 | 0.14 | 0.18 |
| 3011 | 0.69 | 0.14 | 0.17 |
| 3013 | 0.66 | 0.19 | 0.15 |
| 3016 | 0.72 | 0.13 | 0.15 |
| 3017 | 0.73 | 0.11 | 0.16 |
| 3018 | 0.67 | 0.16 | 0.17 |
| 3020 | 0.75 | 0.11 | 0.14 |
| 3021 | 0.7 | 0.14 | 0.16 |
| 3025 | 0.65 | 0.15 | 0.2 |
| 3028 | 0.64 | 0.1 | 0.26 |
| 3029 | 0.71 | 0.12 | 0.17 |
| 3030 | 0.75 | 0.08 | 0.18 |
| 3032 | 0.73 | 0.09 | 0.18 |
| 3033 | 0.69 | 0.1 | 0.22 |
| 3036 | 0.69 | 0.12 | 0.19 |
| 3038 | 0.73 | 0.08 | 0.19 |
| 3039 | 0.62 | 0.13 | 0.26 |
| 3040 | 0.66 | 0.15 | 0.19 |
| 3043 | 0.72 | 0.13 | 0.16 |
| 3047 | 0.57 | 0.13 | 0.3 |
| 3051 | 0.66 | 0.17 | 0.18 |
| 3053 | 0.72 | 0.08 | 0.21 |
| 3056 | 0.69 | 0.11 | 0.21 |
| 3057 | 0.62 | 0.17 | 0.21 |
| 3058 | 0.72 | 0.14 | 0.13 |
| 3060 | 0.72 | 0.1 | 0.18 |
| 3061 | 0.7 | 0.16 | 0.14 |
| 3064 | 0.79 | 0.1 | 0.11 |
| 3067 | 0.67 | 0.12 | 0.21 |
| 3070 | 0.8 | 0.07 | 0.13 |
| 3072 | 0.75 | 0.11 | 0.14 |
| 3073 | 0.73 | 0.08 | 0.19 |
| 3076 | 0.76 | 0.1 | 0.14 |
| 3077 | 0.73 | 0.12 | 0.15 |
| 3078 | 0.72 | 0.11 | 0.17 |
| 3079 | 0.62 | 0.2 | 0.19 |
| 3080 | 0.64 | 0.14 | 0.23 |
| 3084 | 0.66 | 0.12 | 0.22 |
| 3085 | 0.71 | 0.16 | 0.13 |
| 3086 | 0.69 | 0.15 | 0.16 |
| 3087 | 0.62 | 0.22 | 0.16 |
| 3089 | 0.72 | 0.13 | 0.16 |
| 3090 | 0.72 | 0.13 | 0.16 |
| 3091 | 0.72 | 0.11 | 0.17 |
| 3092 | 0.72 | 0.11 | 0.17 |
| 3093 | 0.72 | 0.09 | 0.19 |
| 3095 | 0.67 | 0.15 | 0.18 |
| 3097 | 0.68 | 0.13 | 0.2 |
| 3104 | 0.7 | 0.12 | 0.17 |
| 3105 | 0.73 | 0.11 | 0.16 |
| 3106 | 0.76 | 0.08 | 0.15 |
| 3107 | 0.7 | 0.13 | 0.17 |
| 3108 | 0.68 | 0.1 | 0.22 |
| 3110 | 0.74 | 0.11 | 0.15 |
| 3111 | 0.71 | 0.15 | 0.14 |
| 3115 | 0.69 | 0.11 | 0.2 |
| 3116 | 0.59 | 0.14 | 0.27 |
| 3118 | 0.73 | 0.15 | 0.12 |
| 3120 | 0.61 | 0.17 | 0.23 |
| 3121 | 0.6 | 0.2 | 0.2 |
| 3122 | 0.67 | 0.14 | 0.2 |
| 3123 | 0.69 | 0.14 | 0.18 |
| 3124 | 0.73 | 0.1 | 0.18 |
| 3125 | 0.75 | 0.11 | 0.14 |
| 3129 | 0.71 | 0.11 | 0.17 |
| 3133 | 0.72 | 0.08 | 0.2 |
| 3139 | 0.74 | 0.1 | 0.17 |
| 3142 | 0.66 | 0.12 | 0.22 |
| 3143 | 0.65 | 0.17 | 0.18 |
| 3144 | 0.59 | 0.15 | 0.27 |
| 3146 | 0.72 | 0.13 | 0.16 |
| 3150 | 0.76 | 0.1 | 0.14 |
| 3155 | 0.72 | 0.1 | 0.18 |
| 3156 | 0.7 | 0.11 | 0.19 |
| 3159 | 0.59 | 0.22 | 0.19 |
| 3160 | 0.74 | 0.11 | 0.14 |
| 3161 | 0.73 | 0.13 | 0.15 |
| 3164 | 0.59 | 0.22 | 0.19 |
| 3170 | 0.72 | 0.09 | 0.2 |
| 3171 | 0.63 | 0.19 | 0.18 |
| 3173 | 0.74 | 0.1 | 0.16 |
| 3176 | 0.67 | 0.11 | 0.22 |
| 3178 | 0.67 | 0.13 | 0.2 |
| 3179 | 0.74 | 0.1 | 0.16 |
| 3181 | 0.68 | 0.12 | 0.2 |
| 3183 | 0.71 | 0.12 | 0.17 |
| 3186 | 0.65 | 0.13 | 0.22 |
| 3191 | 0.68 | 0.15 | 0.17 |
| 3192 | 0.71 | 0.14 | 0.16 |
| 3199 | 0.67 | 0.1 | 0.23 |
| 3200 | 0.71 | 0.1 | 0.19 |
| 3201 | 0.64 | 0.13 | 0.23 |
| 3203 | 0.73 | 0.14 | 0.13 |
| 3204 | 0.66 | 0.11 | 0.23 |
| 3205 | 0.76 | 0.09 | 0.15 |
| 3206 | 0.6 | 0.13 | 0.27 |
| 3208 | 0.69 | 0.15 | 0.17 |
| 3209 | 0.64 | 0.15 | 0.22 |
| 3210 | 0.71 | 0.11 | 0.18 |
| 3211 | 0.67 | 0.12 | 0.21 |
| 3212 | 0.62 | 0.16 | 0.22 |
| 3213 | 0.67 | 0.19 | 0.14 |
| 3215 | 0.69 | 0.1 | 0.21 |
| 3218 | 0.61 | 0.17 | 0.22 |
| 3220 | 0.68 | 0.14 | 0.18 |
| 3221 | 0.72 | 0.08 | 0.2 |
| 3222 | 0.74 | 0.1 | 0.16 |
| 3225 | 0.78 | 0.09 | 0.13 |
| 3226 | 0.67 | 0.14 | 0.19 |
| 3228 | 0.72 | 0.1 | 0.18 |
| 3231 | 0.73 | 0.09 | 0.19 |
| 3234 | 0.62 | 0.13 | 0.26 |
| 3235 | 0.52 | 0.21 | 0.27 |
| 3236 | 0.61 | 0.11 | 0.28 |
| 3239 | 0.68 | 0.11 | 0.2 |
| 3240 | 0.71 | 0.13 | 0.16 |
| 3241 | 0.75 | 0.09 | 0.16 |
| 3243 | 0.76 | 0.09 | 0.15 |
| 3244 | 0.68 | 0.1 | 0.22 |
| 3245 | 0.72 | 0.13 | 0.15 |
| 3248 | 0.76 | 0.1 | 0.14 |
| 3250 | 0.74 | 0.1 | 0.16 |
| 3253 | 0.65 | 0.1 | 0.26 |
| 3254 | 0.75 | 0.12 | 0.12 |
| 3255 | 0.72 | 0.11 | 0.18 |
| 3256 | 0.71 | 0.1 | 0.19 |
| 4001 | 0.74 | 0.09 | 0.17 |
| 4002 | 0.58 | 0.11 | 0.32 |
| 4003 | 0.63 | 0.16 | 0.21 |
| 4005 | 0.56 | 0.22 | 0.22 |
| 4017 | 0.59 | 0.18 | 0.24 |
| 4019 | 0.64 | 0.14 | 0.23 |
| 4020 | 0.6 | 0.18 | 0.22 |
| 4021 | 0.65 | 0.19 | 0.16 |
| 4022 | 0.69 | 0.13 | 0.19 |
| 4023 | 0.68 | 0.11 | 0.21 |
| 4024 | 0.53 | 0.24 | 0.23 |
| 4028 | 0.64 | 0.17 | 0.2 |
| 4032 | 0.63 | 0.18 | 0.19 |
| 4037 | 0.64 | 0.17 | 0.19 |
| 4039 | 0.61 | 0.14 | 0.25 |
| 4043 | 0.74 | 0.1 | 0.16 |
| 4046 | 0.65 | 0.13 | 0.22 |
| 4047 | 0.75 | 0.07 | 0.18 |
| 4048 | 0.63 | 0.18 | 0.19 |
| 4049 | 0.61 | 0.26 | 0.14 |
| 4054 | 0.65 | 0.12 | 0.23 |
| 4055 | 0.7 | 0.13 | 0.17 |
| 4056 | 0.59 | 0.11 | 0.29 |
| 4061 | 0.51 | 0.28 | 0.21 |
| 4062 | 0.6 | 0.14 | 0.26 |
| 4065 | 0.6 | 0.13 | 0.27 |
| 4066 | 0.64 | 0.11 | 0.25 |
| 4068 | 0.62 | 0.15 | 0.23 |
| 4069 | 0.67 | 0.12 | 0.21 |
| 4070 | 0.59 | 0.25 | 0.16 |
| 4073 | 0.69 | 0.17 | 0.15 |
| 4076 | 0.71 | 0.15 | 0.14 |
| 4077 | 0.66 | 0.09 | 0.25 |
| 4079 | 0.67 | 0.14 | 0.19 |
| 4084 | 0.73 | 0.09 | 0.18 |
| 4086 | 0.66 | 0.17 | 0.18 |
| 4088 | 0.73 | 0.1 | 0.17 |
| 4090 | 0.75 | 0.1 | 0.14 |
| 4091 | 0.64 | 0.19 | 0.18 |
| 4093 | 0.71 | 0.09 | 0.2 |
| 4094 | 0.67 | 0.12 | 0.22 |
| 4095 | 0.65 | 0.16 | 0.2 |
| 4099 | 0.69 | 0.08 | 0.23 |
| 4100 | 0.56 | 0.18 | 0.26 |
| 4102 | 0.66 | 0.11 | 0.24 |
| 4103 | 0.62 | 0.18 | 0.2 |
| 4105 | 0.66 | 0.16 | 0.18 |
| 4107 | 0.65 | 0.15 | 0.2 |
| 4108 | 0.61 | 0.12 | 0.27 |
| 4111 | 0.73 | 0.1 | 0.17 |
| 4115 | 0.64 | 0.17 | 0.19 |
| 4116 | 0.7 | 0.11 | 0.18 |
| 4119 | 0.74 | 0.09 | 0.16 |
| 4122 | 0.65 | 0.11 | 0.25 |
| 4123 | 0.68 | 0.17 | 0.15 |
| 4126 | 0.58 | 0.18 | 0.25 |
| 4127 | 0.61 | 0.23 | 0.15 |
| 4129 | 0.63 | 0.15 | 0.22 |
| 4130 | 0.74 | 0.1 | 0.16 |
| 4137 | 0.66 | 0.12 | 0.22 |
| 4138 | 0.65 | 0.18 | 0.17 |
| 4142 | 0.69 | 0.12 | 0.19 |
| 4144 | 0.65 | 0.18 | 0.18 |
| 4145 | 0.6 | 0.18 | 0.22 |
| 4146 | 0.74 | 0.11 | 0.14 |
| 4147 | 0.55 | 0.15 | 0.3 |
| 4148 | 0.67 | 0.1 | 0.23 |
| 4149 | 0.72 | 0.14 | 0.14 |
| 4150 | 0.74 | 0.1 | 0.15 |
| 4151 | 0.61 | 0.14 | 0.25 |
| 4152 | 0.63 | 0.17 | 0.2 |
| 4153 | 0.68 | 0.17 | 0.16 |
| 4157 | 0.67 | 0.18 | 0.15 |
| 4162 | 0.65 | 0.12 | 0.23 |
| 4164 | 0.67 | 0.11 | 0.22 |
| 4166 | 0.41 | 0.48 | 0.11 |
| 4167 | 0.65 | 0.11 | 0.24 |
| 4168 | 0.68 | 0.19 | 0.12 |
| 4170 | 0.68 | 0.1 | 0.22 |
| 4171 | 0.62 | 0.13 | 0.26 |
| 4172 | 0.69 | 0.14 | 0.17 |
| 4174 | 0.63 | 0.18 | 0.19 |
| 4175 | 0.68 | 0.13 | 0.19 |
| 4176 | 0.66 | 0.11 | 0.23 |
| 4177 | 0.57 | 0.19 | 0.24 |
| 4180 | 0.71 | 0.1 | 0.19 |
| 4183 | 0.73 | 0.12 | 0.16 |
| 4187 | 0.59 | 0.16 | 0.25 |
| 4188 | 0.63 | 0.15 | 0.23 |
| 4190 | 0.6 | 0.12 | 0.27 |
| 4193 | 0.7 | 0.16 | 0.14 |
| 4195 | 0.59 | 0.17 | 0.24 |
| 4196 | 0.69 | 0.17 | 0.13 |
| 4197 | 0.7 | 0.12 | 0.18 |
| 4198 | 0.71 | 0.12 | 0.18 |
| 4199 | 0.72 | 0.12 | 0.16 |
| 4200 | 0.69 | 0.13 | 0.18 |
| 4202 | 0.69 | 0.16 | 0.15 |
| 4203 | 0.61 | 0.19 | 0.2 |
| 4207 | 0.56 | 0.13 | 0.31 |
| 4208 | 0.63 | 0.2 | 0.16 |
| 4212 | 0.73 | 0.12 | 0.15 |
| 4213 | 0.57 | 0.19 | 0.23 |
| 4215 | 0.65 | 0.19 | 0.16 |
| 4216 | 0.66 | 0.11 | 0.23 |
| 4217 | 0.67 | 0.14 | 0.19 |
| 4221 | 0.64 | 0.14 | 0.22 |
| 4222 | 0.69 | 0.17 | 0.15 |
| 4224 | 0.62 | 0.15 | 0.23 |
| 4226 | 0.5 | 0.17 | 0.33 |
| 4229 | 0.71 | 0.13 | 0.17 |
| 4230 | 0.75 | 0.12 | 0.13 |
| 4231 | 0.52 | 0.37 | 0.12 |
| 4234 | 0.72 | 0.11 | 0.18 |
| 4239 | 0.67 | 0.1 | 0.24 |
| 4241 | 0.6 | 0.2 | 0.21 |
| 4244 | 0.64 | 0.2 | 0.16 |
| 4245 | 0.64 | 0.21 | 0.14 |
| 4248 | 0.66 | 0.15 | 0.19 |
| 4249 | 0.58 | 0.13 | 0.29 |
| 4252 | 0.69 | 0.14 | 0.18 |
| 4257 | 0.63 | 0.11 | 0.26 |
| 4258 | 0.61 | 0.2 | 0.19 |
| 4259 | 0.7 | 0.13 | 0.17 |
| 4261 | 0.68 | 0.14 | 0.18 |
| 4263 | 0.6 | 0.22 | 0.18 |
| 4264 | 0.68 | 0.14 | 0.18 |
| 4265 | 0.69 | 0.14 | 0.17 |
| 4267 | 0.71 | 0.08 | 0.21 |
| 4268 | 0.64 | 0.19 | 0.17 |
| 4271 | 0.68 | 0.15 | 0.17 |
| 4275 | 0.65 | 0.15 | 0.2 |
| 4277 | 0.61 | 0.18 | 0.21 |
| 4278 | 0.67 | 0.13 | 0.2 |
| 4284 | 0.66 | 0.17 | 0.16 |
| 4288 | 0.52 | 0.36 | 0.13 |
| 4289 | 0.45 | 0.45 | 0.1 |
| 4290 | 0.63 | 0.14 | 0.23 |
| 4292 | 0.73 | 0.11 | 0.16 |
| 4293 | 0.7 | 0.1 | 0.2 |
| 4294 | 0.65 | 0.18 | 0.17 |
| 4295 | 0.67 | 0.1 | 0.23 |
| 4296 | 0.63 | 0.15 | 0.22 |
| 4298 | 0.65 | 0.17 | 0.19 |
| 4299 | 0.64 | 0.14 | 0.22 |
| 4301 | 0.75 | 0.11 | 0.14 |
| 4303 | 0.63 | 0.15 | 0.22 |
| 4306 | 0.67 | 0.17 | 0.17 |
| 4310 | 0.46 | 0.42 | 0.12 |
| 4311 | 0.74 | 0.1 | 0.16 |
| 4312 | 0.71 | 0.12 | 0.17 |
| 4313 | 0.67 | 0.15 | 0.18 |
| 4314 | 0.73 | 0.12 | 0.14 |
| 4315 | 0.67 | 0.14 | 0.19 |
| 4317 | 0.7 | 0.16 | 0.14 |
| 4318 | 0.72 | 0.11 | 0.17 |
| 4320 | 0.66 | 0.12 | 0.22 |
| 4322 | 0.68 | 0.12 | 0.2 |
| 4324 | 0.71 | 0.12 | 0.17 |
| 4326 | 0.68 | 0.16 | 0.17 |
| 4329 | 0.65 | 0.16 | 0.18 |
| 4332 | 0.72 | 0.12 | 0.17 |
| 4333 | 0.66 | 0.18 | 0.16 |
| 4334 | 0.62 | 0.13 | 0.25 |
| 4335 | 0.67 | 0.17 | 0.16 |
| 4336 | 0.67 | 0.14 | 0.19 |
| 4337 | 0.64 | 0.12 | 0.24 |
| 4340 | 0.73 | 0.1 | 0.18 |
| 4342 | 0.68 | 0.17 | 0.16 |
| 4343 | 0.69 | 0.16 | 0.15 |
| 4344 | 0.61 | 0.21 | 0.18 |
| 4345 | 0.69 | 0.12 | 0.19 |
| 4347 | 0.68 | 0.1 | 0.22 |
| 4351 | 0.72 | 0.11 | 0.16 |
| 4352 | 0.66 | 0.18 | 0.16 |
| 4353 | 0.66 | 0.17 | 0.16 |
| 4354 | 0.63 | 0.15 | 0.22 |
| 4356 | 0.77 | 0.11 | 0.12 |
| 4359 | 0.69 | 0.13 | 0.19 |
| 4360 | 0.64 | 0.16 | 0.2 |
| 4362 | 0.6 | 0.19 | 0.21 |
| 4366 | 0.68 | 0.15 | 0.17 |
| 4367 | 0.65 | 0.11 | 0.23 |
| 4369 | 0.61 | 0.18 | 0.2 |
| 4370 | 0.69 | 0.19 | 0.12 |
| 4375 | 0.73 | 0.13 | 0.15 |
| 4376 | 0.73 | 0.13 | 0.14 |
| 4377 | 0.72 | 0.1 | 0.18 |
| 4378 | 0.72 | 0.09 | 0.19 |
| 4379 | 0.66 | 0.16 | 0.17 |
| 4380 | 0.66 | 0.14 | 0.21 |
| 4381 | 0.68 | 0.12 | 0.2 |
| 4382 | 0.7 | 0.14 | 0.16 |
| 4383 | 0.71 | 0.11 | 0.19 |
| 4384 | 0.64 | 0.13 | 0.23 |
| 4385 | 0.72 | 0.15 | 0.13 |
| 4386 | 0.59 | 0.22 | 0.19 |
| 4387 | 0.72 | 0.07 | 0.21 |
| 4388 | 0.64 | 0.19 | 0.16 |
| 4389 | 0.67 | 0.15 | 0.18 |
| 4390 | 0.61 | 0.18 | 0.22 |
| 4391 | 0.68 | 0.12 | 0.2 |
| 4392 | 0.67 | 0.14 | 0.2 |
| 4394 | 0.73 | 0.1 | 0.18 |
| 4395 | 0.65 | 0.12 | 0.23 |
| 4397 | 0.65 | 0.11 | 0.24 |
| 4398 | 0.75 | 0.09 | 0.16 |
| 4400 | 0.68 | 0.15 | 0.17 |
| 4401 | 0.59 | 0.19 | 0.22 |
| 4402 | 0.7 | 0.11 | 0.19 |
| 4403 | 0.55 | 0.32 | 0.14 |
| 4404 | 0.71 | 0.12 | 0.18 |
| 4405 | 0.61 | 0.13 | 0.26 |
| 4406 | 0.6 | 0.13 | 0.28 |
| 4408 | 0.69 | 0.16 | 0.15 |
| 4409 | 0.62 | 0.16 | 0.22 |
| 4411 | 0.69 | 0.16 | 0.15 |
| 4412 | 0.73 | 0.1 | 0.17 |
| 4413 | 0.72 | 0.14 | 0.14 |
| 4414 | 0.64 | 0.21 | 0.15 |
| 4415 | 0.61 | 0.15 | 0.24 |
| 4417 | 0.72 | 0.09 | 0.19 |
| 4418 | 0.64 | 0.18 | 0.19 |
| 4420 | 0.63 | 0.15 | 0.23 |
| 4421 | 0.64 | 0.11 | 0.25 |
| 4422 | 0.67 | 0.17 | 0.16 |
| 4423 | 0.69 | 0.14 | 0.18 |
| 4424 | 0.72 | 0.1 | 0.18 |
| 4425 | 0.74 | 0.09 | 0.17 |
| 4426 | 0.67 | 0.14 | 0.19 |
| 4432 | 0.69 | 0.08 | 0.23 |
| 4438 | 0.64 | 0.16 | 0.2 |
| 4439 | 0.65 | 0.14 | 0.2 |
| 4440 | 0.65 | 0.12 | 0.23 |
| 4441 | 0.51 | 0.22 | 0.27 |
| 4443 | 0.58 | 0.3 | 0.13 |
| 4445 | 0.7 | 0.12 | 0.18 |
| 4446 | 0.63 | 0.15 | 0.22 |
| 4447 | 0.68 | 0.14 | 0.19 |
| 5001 | 0.72 | 0.1 | 0.18 |
| 5002 | 0.72 | 0.14 | 0.14 |
| 5003 | 0.64 | 0.14 | 0.22 |
| 5004 | 0.73 | 0.1 | 0.17 |
| 5005 | 0.65 | 0.19 | 0.16 |
| 5006 | 0.74 | 0.11 | 0.16 |
| 5007 | 0.63 | 0.16 | 0.21 |
| 5008 | 0.6 | 0.19 | 0.21 |
| 5009 | 0.74 | 0.1 | 0.16 |
| 5010 | 0.64 | 0.25 | 0.12 |
| 5011 | 0.66 | 0.14 | 0.2 |
| 5012 | 0.67 | 0.16 | 0.17 |
| 5013 | 0.62 | 0.2 | 0.18 |
| 5014 | 0.7 | 0.11 | 0.19 |
| 5015 | 0.67 | 0.1 | 0.23 |
| 5016 | 0.68 | 0.12 | 0.2 |
| 5017 | 0.69 | 0.08 | 0.24 |
| 5018 | 0.71 | 0.07 | 0.22 |
| 5019 | 0.66 | 0.15 | 0.19 |
| 5020 | 0.64 | 0.09 | 0.27 |
| 5021 | 0.61 | 0.17 | 0.23 |
| 5022 | 0.66 | 0.09 | 0.25 |
| 5023 | 0.64 | 0.17 | 0.19 |
| 5024 | 0.72 | 0.08 | 0.21 |
| 5025 | 0.76 | 0.11 | 0.14 |
| 5026 | 0.59 | 0.15 | 0.27 |
| 5027 | 0.62 | 0.11 | 0.27 |
| 5028 | 0.77 | 0.07 | 0.16 |
| 5029 | 0.75 | 0.1 | 0.15 |
| 5030 | 0.66 | 0.09 | 0.26 |
| 5031 | 0.74 | 0.1 | 0.16 |
| 5032 | 0.74 | 0.11 | 0.14 |
| 5033 | 0.66 | 0.17 | 0.17 |
| 5034 | 0.59 | 0.11 | 0.29 |
| 5036 | 0.72 | 0.1 | 0.18 |
| 5037 | 0.63 | 0.07 | 0.29 |
| 5039 | 0.59 | 0.17 | 0.24 |
| 5041 | 0.7 | 0.11 | 0.19 |
| 5042 | 0.61 | 0.16 | 0.23 |
| 5043 | 0.64 | 0.19 | 0.16 |
| 5044 | 0.63 | 0.19 | 0.18 |
| 5045 | 0.68 | 0.12 | 0.21 |
| 5046 | 0.7 | 0.1 | 0.2 |
| 5047 | 0.66 | 0.13 | 0.21 |
| 5048 | 0.6 | 0.2 | 0.2 |
| 5049 | 0.63 | 0.17 | 0.2 |
| 5050 | 0.69 | 0.11 | 0.2 |
| 5052 | 0.7 | 0.14 | 0.17 |
| 5053 | 0.69 | 0.13 | 0.19 |
| 5054 | 0.75 | 0.09 | 0.16 |
| 5055 | 0.61 | 0.12 | 0.27 |
| 5056 | 0.6 | 0.22 | 0.19 |
| 5057 | 0.61 | 0.15 | 0.23 |
| 5058 | 0.61 | 0.12 | 0.28 |
| 5059 | 0.68 | 0.09 | 0.23 |
| 5060 | 0.66 | 0.13 | 0.22 |
| 5061 | 0.6 | 0.2 | 0.21 |
| 5062 | 0.73 | 0.1 | 0.17 |
| 5063 | 0.61 | 0.15 | 0.25 |
| 5064 | 0.63 | 0.16 | 0.22 |
| 5065 | 0.72 | 0.11 | 0.17 |
| 5066 | 0.73 | 0.11 | 0.16 |
| 5067 | 0.7 | 0.12 | 0.18 |
| 5068 | 0.69 | 0.14 | 0.17 |
| 5069 | 0.62 | 0.14 | 0.25 |
| 5071 | 0.64 | 0.18 | 0.18 |
| 5072 | 0.67 | 0.16 | 0.18 |
| 5073 | 0.72 | 0.12 | 0.16 |
| 5074 | 0.61 | 0.2 | 0.19 |
| 5075 | 0.61 | 0.17 | 0.22 |
| 5076 | 0.67 | 0.12 | 0.21 |
| 5077 | 0.7 | 0.16 | 0.15 |
| 5079 | 0.63 | 0.2 | 0.18 |
| 5081 | 0.7 | 0.16 | 0.14 |
| 5082 | 0.67 | 0.1 | 0.23 |
| 5083 | 0.75 | 0.1 | 0.15 |
| 6001 | 0.71 | 0.13 | 0.16 |
| 6005 | 0.62 | 0.17 | 0.22 |
| 6006 | 0.58 | 0.2 | 0.22 |
| 6007 | 0.73 | 0.11 | 0.17 |
| 6008 | 0.75 | 0.11 | 0.13 |
| 6011 | 0.75 | 0.09 | 0.17 |
| 6013 | 0.65 | 0.19 | 0.16 |
| 6014 | 0.65 | 0.1 | 0.25 |
| 6016 | 0.75 | 0.14 | 0.11 |
| 6018 | 0.49 | 0.31 | 0.2 |
| 6019 | 0.69 | 0.1 | 0.2 |
| 6020 | 0.68 | 0.12 | 0.21 |
| 6022 | 0.59 | 0.25 | 0.16 |
| 6024 | 0.71 | 0.11 | 0.18 |
| 6028 | 0.59 | 0.26 | 0.14 |
| 6030 | 0.72 | 0.13 | 0.15 |
| 6031 | 0.6 | 0.21 | 0.19 |
| 6032 | 0.66 | 0.19 | 0.15 |
| 6033 | 0.73 | 0.1 | 0.17 |
| 6034 | 0.67 | 0.11 | 0.22 |
| 6035 | 0.74 | 0.09 | 0.18 |
| 6036 | 0.78 | 0.1 | 0.12 |
| 6037 | 0.78 | 0.08 | 0.14 |
| 6038 | 0.56 | 0.17 | 0.27 |
| 6039 | 0.59 | 0.18 | 0.23 |
| 6040 | 0.66 | 0.11 | 0.24 |
| 6041 | 0.64 | 0.12 | 0.24 |
| 6042 | 0.61 | 0.17 | 0.22 |
| 6043 | 0.75 | 0.1 | 0.15 |
| 6044 | 0.71 | 0.09 | 0.2 |
| 6045 | 0.71 | 0.1 | 0.19 |
| 6046 | 0.64 | 0.13 | 0.23 |
| 6047 | 0.74 | 0.07 | 0.19 |
| 6048 | 0.65 | 0.19 | 0.16 |
| 6049 | 0.73 | 0.12 | 0.16 |
| 6050 | 0.59 | 0.18 | 0.24 |
| 6052 | 0.68 | 0.2 | 0.12 |
| 6058 | 0.58 | 0.17 | 0.25 |
| 6059 | 0.62 | 0.1 | 0.28 |
| 6062 | 0.65 | 0.17 | 0.19 |
| 6063 | 0.71 | 0.13 | 0.17 |
| 6064 | 0.69 | 0.14 | 0.18 |
| 6065 | 0.6 | 0.26 | 0.14 |
| 6066 | 0.7 | 0.15 | 0.15 |
| 6067 | 0.7 | 0.14 | 0.16 |
| 6068 | 0.66 | 0.12 | 0.21 |
| 6069 | 0.69 | 0.12 | 0.2 |
| 6070 | 0.69 | 0.16 | 0.15 |
| 6071 | 0.68 | 0.13 | 0.18 |
| 6072 | 0.69 | 0.14 | 0.17 |
| 6073 | 0.65 | 0.19 | 0.16 |
| 6074 | 0.66 | 0.14 | 0.2 |
| 6075 | 0.69 | 0.17 | 0.14 |
| 6076 | 0.7 | 0.1 | 0.2 |
| 6077 | 0.68 | 0.15 | 0.17 |
| 7002 | 0.74 | 0.1 | 0.16 |
| 7003 | 0.69 | 0.09 | 0.22 |
| 7004 | 0.63 | 0.18 | 0.19 |
| 7005 | 0.64 | 0.12 | 0.25 |
| 7007 | 0.74 | 0.11 | 0.15 |
| 7008 | 0.61 | 0.15 | 0.24 |
| 7009 | 0.69 | 0.08 | 0.22 |
| 7010 | 0.64 | 0.18 | 0.18 |
| 7014 | 0.64 | 0.15 | 0.21 |
| 7015 | 0.62 | 0.17 | 0.22 |
| 7017 | 0.66 | 0.12 | 0.22 |
| 7018 | 0.61 | 0.18 | 0.22 |
| 7022 | 0.73 | 0.13 | 0.15 |
| 7024 | 0.73 | 0.11 | 0.16 |
| 7025 | 0.73 | 0.1 | 0.17 |
| 7027 | 0.68 | 0.12 | 0.2 |
| 7033 | 0.64 | 0.1 | 0.26 |
| 7035 | 0.7 | 0.11 | 0.19 |
| 7036 | 0.59 | 0.18 | 0.23 |
| 7037 | 0.64 | 0.1 | 0.26 |
| 7038 | 0.68 | 0.13 | 0.19 |
| 7039 | 0.67 | 0.15 | 0.17 |
| 7040 | 0.67 | 0.14 | 0.19 |
| 7042 | 0.75 | 0.1 | 0.15 |
| 7043 | 0.68 | 0.11 | 0.22 |
| 7045 | 0.75 | 0.1 | 0.16 |
| 7046 | 0.75 | 0.11 | 0.14 |
| 7048 | 0.74 | 0.1 | 0.16 |
| 7049 | 0.7 | 0.17 | 0.13 |
| 7050 | 0.7 | 0.13 | 0.18 |
| 7051 | 0.67 | 0.14 | 0.19 |
| 7053 | 0.75 | 0.11 | 0.15 |
| 7054 | 0.67 | 0.16 | 0.17 |
| 7055 | 0.65 | 0.13 | 0.22 |
| 7058 | 0.66 | 0.1 | 0.24 |
| 7060 | 0.59 | 0.15 | 0.26 |
| 7061 | 0.68 | 0.13 | 0.19 |
| 7062 | 0.7 | 0.09 | 0.2 |
| 7063 | 0.61 | 0.13 | 0.25 |
| 7066 | 0.68 | 0.15 | 0.17 |
| 7067 | 0.68 | 0.15 | 0.17 |
| 8001 | 0.72 | 0.09 | 0.19 |
| 8002 | 0.69 | 0.1 | 0.21 |
| 8004 | 0.71 | 0.13 | 0.16 |
| 8005 | 0.66 | 0.12 | 0.23 |
| 8006 | 0.63 | 0.09 | 0.28 |
| 8008 | 0.62 | 0.2 | 0.18 |
| 8010 | 0.59 | 0.18 | 0.23 |
| 8013 | 0.68 | 0.1 | 0.23 |
| 8015 | 0.79 | 0.08 | 0.13 |
| 8016 | 0.63 | 0.12 | 0.25 |
| 8017 | 0.67 | 0.13 | 0.2 |
| 8018 | 0.53 | 0.24 | 0.23 |
| 8019 | 0.67 | 0.1 | 0.23 |
| 8020 | 0.64 | 0.13 | 0.23 |
| 8021 | 0.73 | 0.14 | 0.13 |
| 8022 | 0.66 | 0.16 | 0.19 |
| 8023 | 0.71 | 0.17 | 0.13 |
| 8024 | 0.82 | 0.07 | 0.12 |
| 8025 | 0.66 | 0.13 | 0.21 |
| 8026 | 0.76 | 0.09 | 0.15 |
| 8027 | 0.64 | 0.17 | 0.19 |
| 8028 | 0.75 | 0.08 | 0.17 |
| 8030 | 0.67 | 0.12 | 0.21 |
| 8031 | 0.67 | 0.15 | 0.19 |
| 8033 | 0.71 | 0.14 | 0.15 |
| 8035 | 0.76 | 0.09 | 0.15 |

* Individual Ancestral Composition= European + African+ Amerindian = 1

**Figure S1. Individual ancestral composition**

Bar graph showing the percentage of genetic ancestry from each sample. The numbers represent the ID of each sample. Green represents Amerindian, red represents African and blue represents European
